# Gut microbiota-derived trimethylamine N-Oxide: a novel target for the treatment of preeclampsia

**DOI:** 10.1080/19490976.2024.2311888

**Published:** 2024-02-13

**Authors:** Jiayi Wang, Yajie Gao, Shuaijun Ren, Jialin Li, Siqian Chen, Jiating Feng, Bing He, Yuping Zhou, Rongrong Xuan

**Affiliations:** a Gynaecology and obstetrics, The First Affiliated Hospital of Ningbo University, Ningbo, China; b Ningbo Key Laboratory of Translational Medicine Research on Gastroenterology and Hepatology, The First Affiliated Hospital of Ningbo University, Ningbo, China; c Health Science Center, Ningbo University, Ningbo, China

**Keywords:** Preeclampsia, gut microbiota, TMAO, placental trophoblast cells, vascular endothelial cells

## Abstract

Pre-eclampsia (PE) is the most common complication of pregnancy and seriously threatens the health and safety of the mother and child. Studies have shown that an imbalance in gut microbiota can affect the progression of PE. Trimethylamine N-oxide (TMAO) is an intestinal microbiota-derived metabolite that is thought to be involved in the occurrence of PE; however, its causal relationship and mechanism remain unclear. In this clinical cohort study, including 28 patients with eclampsia and 39 matched healthy controls, fecal samples were collected for 16S rRNA gene sequencing, and serum was collected for targeted metabolomics research. The results showed that the level of TMAO and the abundance of its source bacteria had significantly increased in patients with PE, and were positively correlated with the clinical progression of PE. Fecal microbiota transplantation (FMT) was applied to an antibiotic-depleted-treated mouse model and targeted inhibition of TMAO. The results of the FMT experiment revealed that mice that received fecal microbiota transplantation from patients with PE developed typical PE symptoms and increased oxidative stress and inflammatory damage, both of which were reversed by 3,3-Dimethyl-1-butanol (DMB), a TMAO inhibitor, which also improved pregnancy outcomes in the model mice. Similar results were obtained in the classical NG-Nitroarginine methyl ester (L-NAME) induced PE mouse model. Mechanistically, TMAO promotes the progression of PE by regulating inflammatory and oxidative stress-related signaling pathways, affecting the migration and angiogenesis of vascular endothelial cells, as well as the migration and invasion of trophoblast cells. Our results reveal the role and mechanism of gut microbiota and TMAO in the progression of PE, provides new ideas for exploring the pathogenesis and therapeutic targets of PE, and determines the potential application value of TMAO as a target for PE intervention.

## GRAPHICAL ABSTRACT

**Figure:**
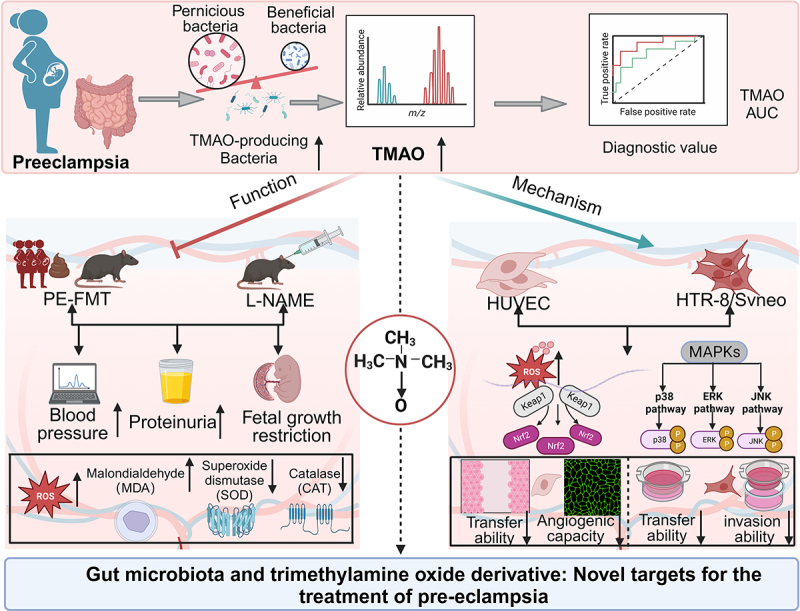

## Introduction

Pre-eclampsia (PE) is a complex multisystem disease unique to pregnancy, with a global incidence of approximately 2%–8%.^1^ PE, which is characterized by hypertension and end-organ dysfunction after 20 weeks of gestation, is associated with an increased risk of adverse pregnancy outcomes, such as preterm birth, fetal growth restriction, and stillbirth, and it is one of the main causes of maternal and perinatal mortality.^2–4^ At present, the clinical treatment of PE is mainly symptomatic, including the use of antispasmodics, antihypertensives, and sedation; however, these strategies cannot fundamentally change and correct the cause of PE, and do not have a preventive effect. Moreover, often only termination of pregnancy is offered as a “solution” to PE, resulting in poor maternal and neonatal prognoses.^5^ Therefore, it is crucial to seek new targets and explore new intervention methods.

The etiology and pathogenesis of PE are complex and have not yet been fully elucidated. Redman’s “two-stage theory” highlights that the invasion ability of extravillous trophoblasts in PE is impaired, resulting in “superficial placental implantation” and uterine spiral artery recasting, leading to placental ischemia, hypoxia, and oxidative stress.^6^ Ultimately, this process mediates the release of a variety of placental factors into the maternal circulation, contributing to the systemic inflammatory response, systemic small vessel spasm, and endothelial cell damage, thereby causing damage to multiple organs. The intestinal flora plays an important role in maintaining the health of the body, with functions including maintaining the intestinal mucosal barrier and regulating metabolism and immunity.^7^ In recent years, several studies have shown that changes in the gut microbiome are associated with pregnancy outcomes, compared to healthy pregnant women, the diversity of intestinal flora in patients with PE is reduced, and the composition and structure are significantly different. Indeed, the imbalance of intestinal flora is an important factor leading to PE.^3,8,9^ However, the causal relationship between intestinal flora imbalance and PE and its mechanism need to be further discussed, and the key flora affecting the progression of PE remain unclear. Metabolites are an important pathway through which the gut microbiota plays a role in human physiology and pathology. Trimethylamine N-oxide (TMAO) is a key metabolite of intestinal flora. Some intestinal flora can produce trimethylamine lyase, which converts alkaloids, such as choline, L-carnitine, and betaine, to trimethylamine (TMA), which is oxidized to TMAO by flavin monooxygenases (FMOs).^10^ Recent studies have shown that TMAO is a potential risk factor for cardiovascular diseases such as hypertension.^11,12^ A previous clinical case – control study showed that both fecal and plasma TMAO concentrations were higher in patients with PE than in healthy controls, suggesting that TMAO is involved in the development of PE.^13^ However, the mechanism of TMAO in the pathogenesis of PE remains unclear. Exploring the associated flora of TMAO sources and the mechanism by which TMAO induces PE may bring new ideas to develop appropriate treatments for PE.

In this clinical case-control study, we used mouse fecal microbiota transplantation (FMT), human umbilical vein endothelial cell (HUVEC), and human placental trophoblast cell (HTR-8/SVneo) oxidative stress models to explore the changes in gut microbiota and its metabolite TMAO in the progression of PE. The aim of this study was to establish the value and potential of TMAO as an intervention target for the treatment of PE, as well as to provide a new target for the prevention and treatment of PE.

## Results

### Gut microbiota of patients with PE shows significant dysregulation

**Figure 1.**
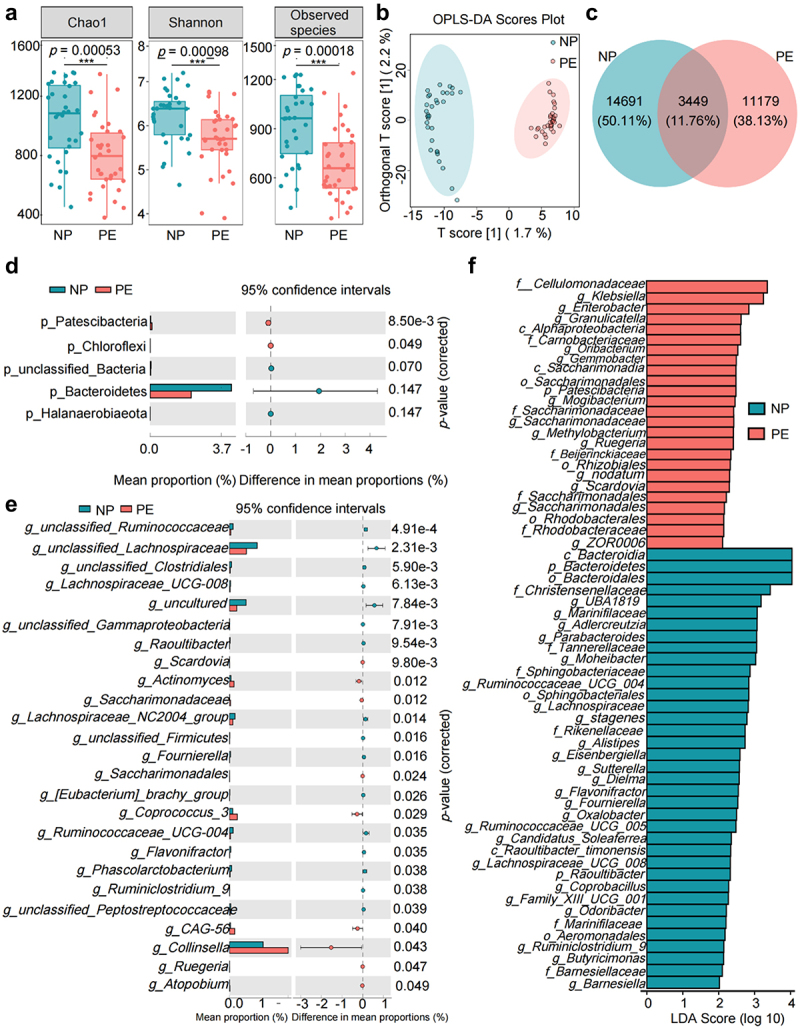
Dysbiosis of gut microbiota in patients with preeclampsia (PE). (a) Comparison of the α diversity of the gut microbiota between the preeclampsia (PE) and normal pregnant women (NP) groups. (b) Comparison of the β diversity of gut microbiota between the PE and NP groups. (c) Venn diagram of operational taxonomic units (OTUs) in the PE and NP groups. (d) Differences in the structure and Amplicon sequence variant (ASV)/OTU ratio of gut microbiota at the phylum level between the PE and NP groups. (e) Differences in the structure and ASV/OTU ratio of gut microbiota at the genus level between the PE and NP groups. (f) Bar plot of the linear discriminant analysis (LDA) value distribution of significantly different species, showing the significantly enriched species and their importance degree in the two groups. Data are presented as the median and quartile in panel A, and statistical analysis was performed using the *t*-test. *p < .05, **p < .01, ***p < .001. NP group: *n* = 29; PE group: *n* = 38.

The results of 16S rRNA gene sequencing technology showed that the Alpha diversity index indicated that both the richness and diversity of gut microbiota in patients with PE were significantly lower than those in NP (Figure 1a), and a significant separation between PE and Normal pregnant women (NP) samples was observed in the supervised orthogonal projections to latent structures discriminant analysis (OPLS-DA) score plots (R2Y = 0.803, Q2 = 0.824) (Figure 1b). Additionally, the community composition of the PE group was significantly changed compared to that of the NP group (Figure 1c). These results suggest that the gut microbiota of patients with PE is generally unbalanced. Moreover, the phyla Patescibacteria and Chloroflexi were significantly more abundant in the PE group than in the NP group (Figure 1d). Additionally, we observed significant differences in the abundance of 25 genera between the PE and NP groups, and the abundance of *Ruegeria* was found to have increased in the PE group (Figure 1e). Next, linear discriminant analysis effect size (LEfSe) analysis was performed to explore the differential flora between the PE and NP groups (LDA >2). The results showed that 62 taxa (37 in the NP group and 25 in the PE group) were identified as potential flora markers, which made a large contribution to the difference between the PE and NP groups (Figure 1f). The above results proved that the diversity and structure of the fecal gut microbiota in patients with PE changed significantly, indicating the important role of fecal gut microbiota in the progression of PE. Notably, we also found an abundance of some bacterial flora in the placentas of patients with PE, which was significantly different from that of the NP group, suggesting that changes in bacterial flora are not only a characterization of disease, but may also be the cause of pregnancy disorders (Supplementary Fig. S1).

### Source of flora and changes in TMAO and its precursors in patients with PE

**Figure 2.**
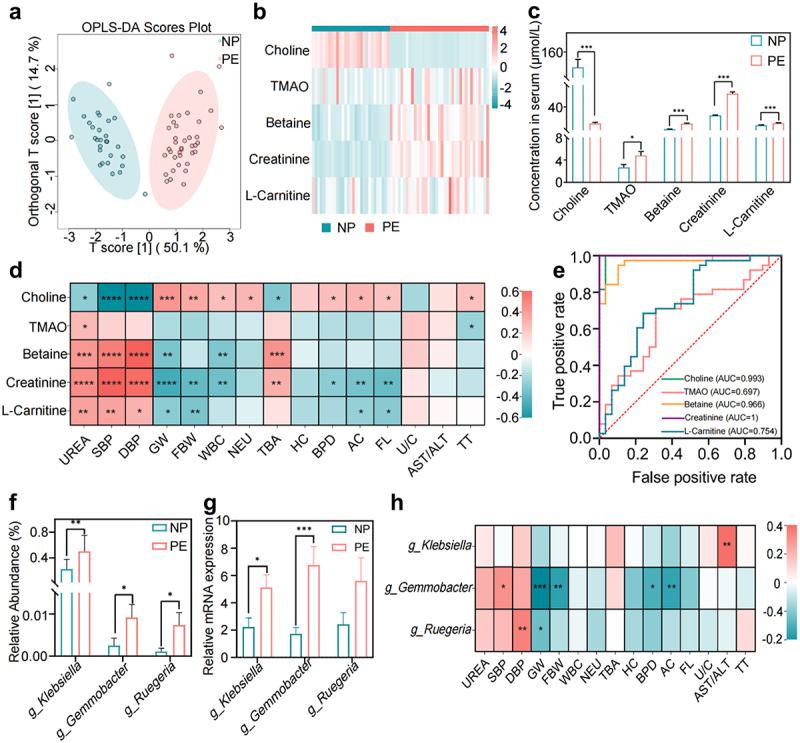
Changes in Trimethylamine N-oxide (TMAO) and its precursors in patients with PE, and source analysis of the microbiota. (a) Differences in TMAO and its precursor levels between the PE and NP groups by orthogonal partial least squares discriminant analysis (OPLS-DA). (b) Heat map of serum TMAO and its precursor expression levels in the PE and NP groups. (c) Quantitative analysis of serum TMAO and its precursors in the NP and PE groups. (d) Correlation analysis between serum TMAO and its precursors and key clinical indicators of PE. (e) The ability of TMAO and its precursors to diagnose PE was analyzed by receiver operating characteristic (ROC) curve. (f) Difference in the abundance of TMAO-producing bacteria between the NP and PE groups. (g) The relative abundance of *klebsiella, Gemmobacter*, and *Ruegeria* of the PE and NP groups. (h) Correlation analysis between TMAO-producing bacteria and PE clinical indicators. Data are presented as the mean ± SD in C and F plots, and statistical analysis was performed using the *t*-test. *p < .05, **p < .01, ***p < .001. NP group: *n* = 29; PE group: *n* = 38. SBP: systolic blood pressure, DBP: diastolic blood pressure, GW: gestational weeks, FBW: fetal birth weight, WBC: white blood cell, NEU: neutrophils, TBA: total bile acid, HC: head circumference, BPD: biparietal diameter, AC: abdominal circumference, FL: femoral length, U/C: Urea/creatinine, AST/ALT: aspartate aminotransferase/alanine aminotransferase, TT: thrombin time.

To investigate the changes and clinical significance of TMAO and its precursors in patients with PE, we next performed targeted metabolomics analysis of serum samples from the PE and NP groups. OPLS-DA (R2Y = 0.886, Q2 = 0.864) showed significant differences in serum TMAO and its precursors between the PE and NP groups (Figure 2a). The results of quantitative analysis showed that serum levels of TMAO and its precursors, betaine, creatinine, and L-carnitine, were significantly increased, but the choline levels were significantly decreased in patients with PE compared to the NP group (Figure 2b–c). The results of correlation analysis of TMAO and its precursor levels with clinical indicators of PE showed that betaine, creatinine, and L-carnitine were significantly positively correlated with blood pressure (including systolic and diastolic blood pressure) and urea/creatinine levels, while betaine was negatively correlated with neonatal birth weight (Figure 2d). These results suggest that pre-TMAO levels are significantly associated with PE symptoms and adverse pregnancy outcomes. The ability of TMAO and its precursors to diagnose PE was also evaluated. Receiver operating characteristic curve (ROC) analysis showed that the area under curve (AUC) of TMAO and its precursors reached 0.7. Among them, creatinine had the highest sensitivity and specificity, with an AUC value of 1 (Figure 2e), suggesting its potential as a new diagnostic marker for PE.

To further understand the relationship between TMAO and gut microbiota, we reviewed KEGG, BIOML, CGR, HBC, GMrepo, and other existing databases, as well as published literature,^14,15^ Metagenomic deep sequencing data were used to construct the traceability table of intestinal flora producing relevant metabolites for traceability analysis. The results showed that the main TMAO-producing bacteria were *Klebsiella, Gemmobacter, Ruegeria*, and other genera (see Supplementary Table S1). The results of 16S rRNA sequencing showed that these TMAO-producing genera were significantly increased in the feces of patients with PE compared to those of NP patients (Figure 2f). In particular, the significant increase in *Klebsiella, Gemmobacter* and *Ruegeria* in the feces of PE group were confirmed by qPCR (Figure 2g). Correlation analysis showed that the abundance of *Gemmobacter* and *Ruegeria* was significantly and positively correlated with systolic and diastolic blood pressure levels, respectively (Figure 2h). The consistency between the changes in TMAO content and the changes in the source flora suggests that the changes in TMAO content may be related to the changes in the source flora.

### PE-FMT induces a PE phenotype in mice that is reversed by TMAO inhibitors

**Figure 3.**
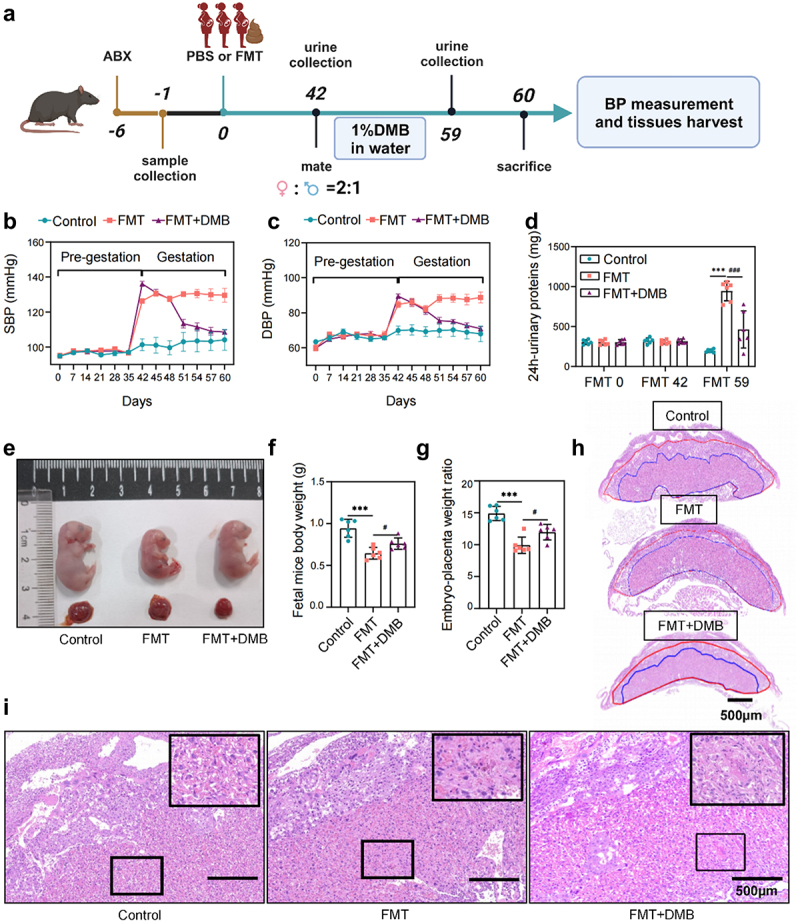
TMAO inhibitor 3,3-dimethyl-1-butanol (DMB) reversed the PE phenotype in mice that received fecal microbiota transplantation from patients with PE. (a) PE-fecal microbiota transplantation (FMT) animal experiment protocol. (b) Dynamic changes and comparison of the systolic blood pressure (SBP) in each group of mice (*n* = 6). (c) Dynamic changes and comparison of the diastolic blood pressure (DBP) among groups (*n* = 6). (d) Twenty-four-hour urinary protein content before FMT, on day 42 of FMT (before pregnancy), and on day 59 of FMT (day 17 of pregnancy). (e) Whole body photograph of embryo and placenta. (f) Weight of fetal mice. (g) Placental efficiency was assessed by the ratio of embryo to placental weight. (h) Representative images of sagittal tissue sections in hematoxylin-eosin (h&e) stained placental tissue. Regions are marked and represented by maze and junctional zones. (i) Representative H&E staining images of the placental maze area of mice in each group. Data are presented as the mean ± SEM. Significant differences based on one-way ANOVA and Tukey’s multiple tests: *p < .05, **p < .01, ***p < .001, control group compared to the FMT group; ^###^*p* < .001, ^##^*p* < .01, ^#^*p* < .05, FMT+DMB group compared to the FMT group.

To assess the causal relationship between gut microbiota dysbiosis and PE, we transplanted fecal samples from patients with PE into mice as donors (Figure 3a). After the mice had been pretreated with broad-spectrum antibiotics to deplete the gut microbiota (Supplemental Figure S2a – c), the recipient mice were gavaged with fecal microbiota from patients with PE, and the control mice were gavaged with an equal volume of sterile phosphate buffered saline (PBS). After the experiment, the cecum was taken to analyze the gut microbiota of the recipient mice by 16S rRNA sequencing. Overall, the recipient mice replicated most of the characteristics of the microbiota from the PE patient donors, and many microbial species, showed similar changes between the recipient mice and the PE patient donors, suggesting successful colonization of the gut microbiota (Supplemental Figure 2d). On day 42 of FMT (before pregnancy), the systolic blood pressure (SBP) of recipient mice was significantly higher than that of control mice, but there was no significant difference in 24 h urinary protein content. However, the blood pressure of the FMT group continued to increase from days 42 to 60 (days 1–18 of pregnancy), and on day 59 of FMT (day 17 of pregnancy), the urinary protein content increased significantly, showing the typical clinical phenotype of PE (Figure 3b–d). Additionally, the size and weight of the fetuses and placentas were significantly reduced in the FMT group compared to those in the control group (Figure 3e–g). To determine the effect of fecal microbiota from patients with PE on placental pathology, we examined the structure of the placental implantation site by comparing the results of histological staining of placental samples from the three groups. The results of hematoxylin-eosin (H&E) staining showed that the ratio between the two functional areas of the placenta (labyrinth and junctional zone) was elevated in the FMT group mice, and the placental infarct size and interstitial collagen deposition in the placental labyrinth layer were increased (Figure 3h–i). We transplanted fecal bacteria from NP into mice, and the results showed that compared to the control group, there was no significant change in the phenotype of the mice, and no toxic reaction (Supplementary Fig. S3).

Co-treatment of FMT mice with 3,3-Dimethyl-1-butanol (DMB), a TMAO inhibitor, significantly reversed hypertension (Figure 3b, c), urinary protein (Figure 3d), and intrauterine growth restriction (Figure 3e) induced by PE fecal microbiota transplantation while increasing the fetal mice weight and the fetal to placental weight ratio (Figure 3f–g). Additionally, the placental vascular hypoperfusion in the FMT group was characterized by infarction foci and low perivascular lymphocyte infiltration, and the vascular hypoperfusion was significantly improved after DMB intervention, with almost no infarction, the DMB intervention group partially restored the pathological changes of PE, and the morphology of the placental labyrinth was similar to that of the control group (Figure 3h–i). These results suggest that an imbalance of gut microbiota may cause the occurrence and development of PE through its key metabolite TMAO. This hypothesis was supported by 16S rRNA sequencing and TMAO-targeted metabolome analysis. Targeted intervention with TMAO could significantly improve the intestinal dysbiosis in the FMT group (Supplementary Fig. S4A-F). The level of TMAO in the FMT group significantly increased compared to that in the control group, while the level of TMAO decreased significantly after targeted inhibition of TMAO (Supplementary Fig. S4G). The results of correlation analysis of TMAO and its precursor levels with clinical indicators of PE mice showed that TMAO were significantly positively correlated with blood pressure (including systolic and diastolic blood pressure) and urinary protein levels, while betaine was negatively correlated with fetal mice weight (Supplementary Fig. S4H).

### PE-FMT promoted oxidative stress and inflammation, which were reversed by TMAO inhibitors

**Figure 4.**
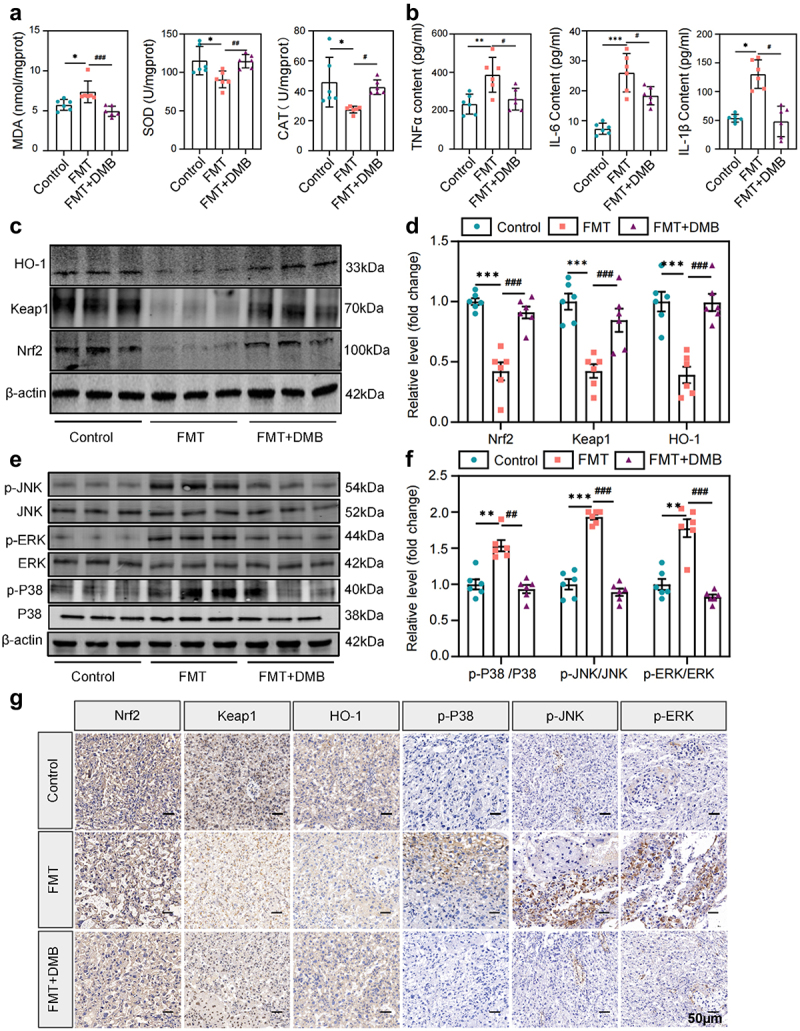
Targeting TMAO significantly attenuated oxidative stress and inflammatory injury in PE-FMT recipient mice. (a) Activity levels of placental oxidative stress injury markers in each group of mice after PE fecal microbiota transplantation. (b) Comparison of serum inflammatory factors (IL-6, TNF-α, IL-1β) levels. (c-d) Western blot was used to detect the expression levels of Nrf2-Keap1 signaling pathway-related molecules in the placental tissues of mice in each group. (e-f) Western blot was used to detect the expression of MAPK family kinases (P38, p-P38, ERK, p-ERK, JNK, p-JNK) signaling pathway-related molecules in the placental tissues of each group. (g) Immunohistochemistry was used to detect the expression levels of MAPK family kinases (p-P38, p-ERK, p-JNK) and Nrf2-Keap1 signaling pathway-related molecules in placental tissues of each group. Data are presented as the mean ± SEM. Significant differences based on one-way ANOVA and Tukey’s multiple tests: *p < .05, **p < .01, ***p < .001, control group compared to the FMT group; ^###^*p* < .001, ^##^*p* < .01, ^#^*p* < .05, FMT+DMB group compared to the FMT group *n* = 6.

Oxidative stress and inflammation play important roles in the pathogenesis of PE.^16,17^ Therefore, we investigated whether fecal microbiota transplantation from patients with PE could cause oxidative stress and inflammatory responses in mouse placentae. The results showed that PE fecal receptor (PE-FMT) mice had noticeable placental oxidative stress damage, as indicated by the increased contents of malondialdehyde (MDA) and decreased activity of antioxidant enzymes such as superoxide dismutase (SOD) and catalase (CAT), whereas the placental oxidative stress level of mice in the FMT group was significantly reduced after targeted TMAO intervention (Figure 4a). In addition to the elevated levels of oxidative stress, compared to the control mice, the PE fecal recipient mice had significantly higher serum levels of inflammatory factors, including interleukin (IL)-6, tumor necrosis factor (TNF)-α, and IL-1β, which were partially reversed by targeted TMAO intervention (Figure 4b). Western blot results showed that placental expression levels of antioxidant proteins, including Nrf2, Keap1, and HO-1 were significantly reduced in PE fecal recipient mice (Figure 4c–d). Additionally, the phosphorylation levels of MAPKs and other inflammatory signaling pathways (p-P38, p-ERK, p-JNK) were significantly increased (Figure 4e–f), indicating the activation of the MAPK/Nrf2-Keap1 pathway, which could be blocked by the addition of the TMAO inhibitor. Similar results were obtained by immunohistochemical testing of key proteins (Figure 4g). Taken together, these results suggest that targeted inhibition of TMAO may have a significant intervention effect on FMT-induced placental oxidative stress and inflammation in mice, and that TMAO is involved in the mechanism by which gut microbiota leads to PE progression.

### Targeted inhibition of TMAO significantly alleviates the clinical phenotype and adverse pregnancy outcomes of PE in the L-NAME mouse model

**Figure 5.**
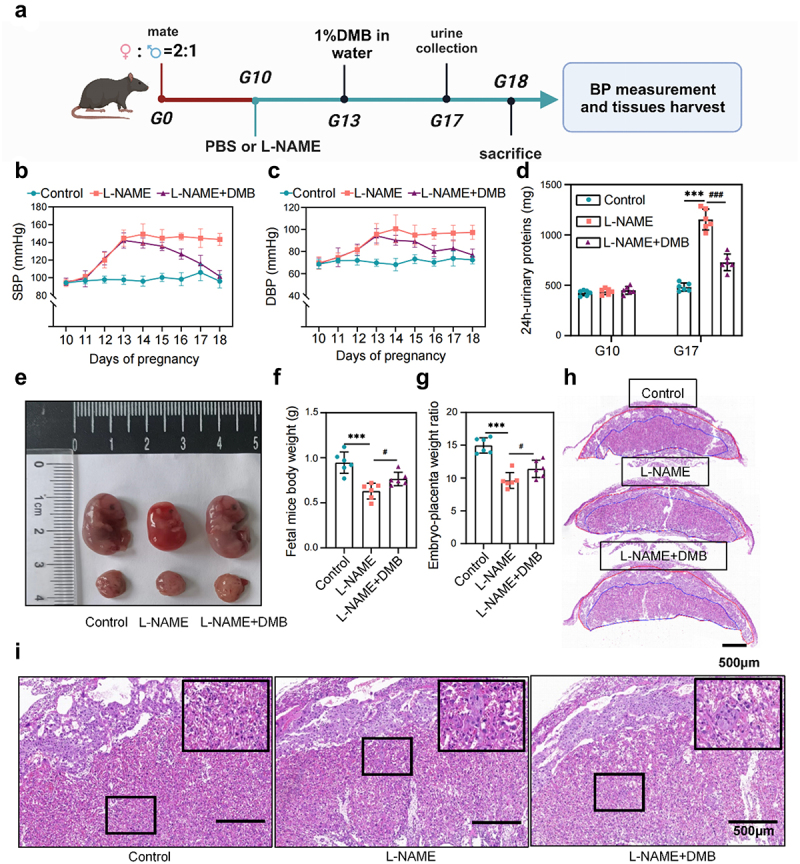
Targeted intervention with TMAO reduces the clinical phenotype of NG-Nitroarginine methyl ester (L-NAME)-induced PE. (a) Animal experimental protocol of L-NAME-induced PE model mice. (b) Dynamic changes and comparison of the SBP in each group during pregnancy (*n* = 6). (c) Dynamic changes and comparison of the DBP in each group during pregnancy (*n* = 6). (d) The urine of G10 and G17 mice in each group was collected, and the urinary protein concentration and 24-h urinary protein content were measured. (e) Whole-body photographs of fetal mice and placentas of the three groups. (f) Weight of the fetal rats. (g) The placental efficiency of the three groups was assessed by the ratio of fetal to placental weight. (h) Representative images of placental tissues stained with H&E. Regions are marked and represented by maze and junctional zones. (i) Representative H&E staining images of the placental maze area of each group of mice. Data are presented as the mean ± SEM. Significant differences based on one-way ANOVA and Tukey’s multiple tests: *p < .05, **p < .01, ***p < .001, control group compared to the L-NAME group; ^###^*p* < .001, ^##^*p* < .01, ^#^*p* < .05, L-NAME+DMB group compared to the L-NAME group.

To confirm the clinical potential of TMAO as an intervention target, we established a classical PE mouse model by subcutaneous injection of NG-Nitroarginine methyl ester (L-NAME) (Figure 5a) and used DMB to target TMAO inhibition to observe its effect on PE progression. We observed the effects of DMB on normal mice in advance and found no adverse results, which rules out the effects of DMB (Supplementary Fig. S5). The systolic and diastolic blood pressures of the PE-model mice were significantly increased on day 13 of pregnancy, indicating successful establishment of the PE model. Blood pressure (Figure 5b–c) and urinary protein levels were significantly reduced (Figure 5d) in PE pregnant rats after DMB intervention. Additionally, the fetal size and weight were significantly lower in the L-NAME group than in the control group, whereas DMB intervention ameliorated fetal growth restriction, with a corresponding increase in the placental/fetal weight ratio in the DMB group compared to the L-NAME group (Figure 5e–g). Histological analysis of the placenta showed an increased ratio between the two functional areas (labyrinth and junction area) of the placenta in the L-NAME group (Figure 5h). We also observed significant differences in placental labyrinth morphology between the L-NAME and control groups (Figure 5i), as shown by increased placental infarct size and interstitial collagen deposition in the placental labyrinth layer. After DMB intervention, the placental pathology improved, and the morphology of the labyrinth was similar to that of the control group. The above results indicate that targeted reduction of TMAO levels significantly reduce hypertension and improve the pregnancy outcome and placental pathological state in the L-NAME-induced PE mouse model, although the specific mechanism remains unclear. The results of 16S rRNA sequencing and TMAO-targeted metabolome analysis showed that targeted intervention in TMAO significantly improved the intestinal dysbiosis in the L-NAME group (Supplementary Fig. S6A – F). Additionally, the level of TMAO in the L-NAME group significantly increased compared to that in the control group, while the level of TMAO decreased significantly after targeted inhibition of TMAO (Supplementary Fig. S6g).

### Targeted inhibition of TMAO significantly improves placental oxidative stress and inflammation in the L-NAME model

**Figure 6.**
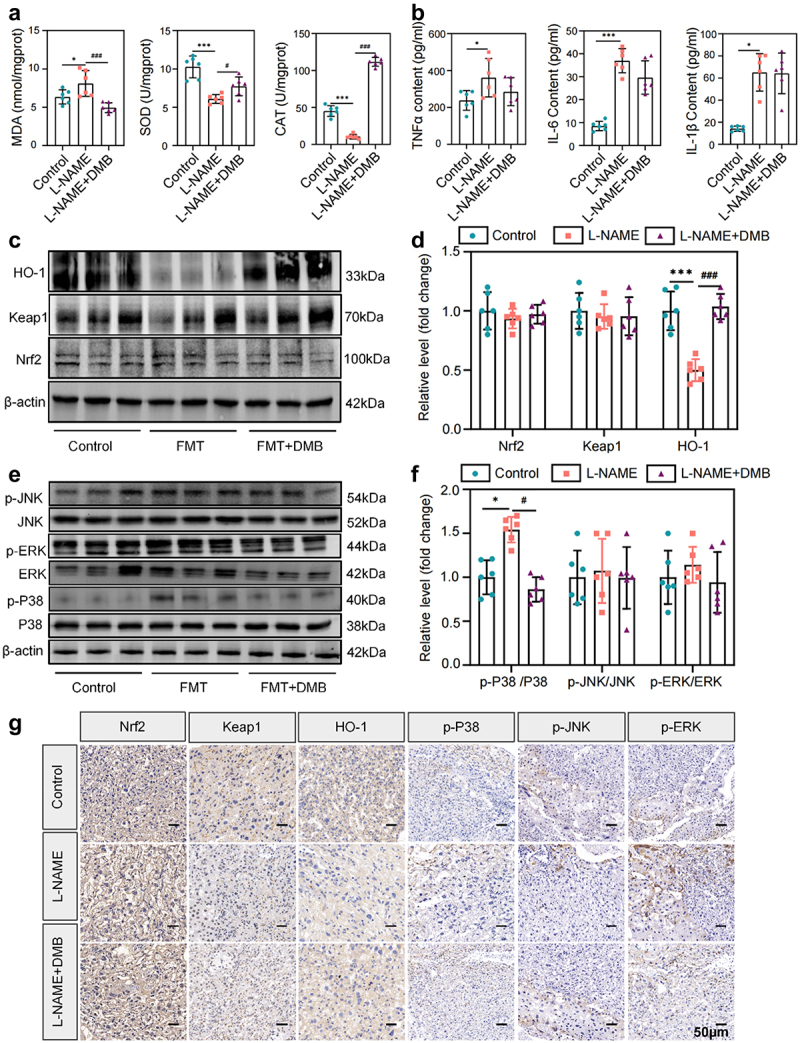
Targeted intervention with TMAO attenuated oxidative stress and inflammation in L-NAME-induced PE mice. (a) Activity levels of placental oxidative stress injury markers in each group of mice. (b) Comparison of serum inflammatory factor (IL-6, TNF-α, IL-1β) levels. (c–d) expression levels of Nrf2-Keap1 signaling pathway-related molecules in placental tissues. (e–f) expression levels of MAPK family kinase (P38, p-P38, ERK, p-ERK, JNK, p-JNK) signaling pathway-related molecules in placental tissues. (g) Immunohistochemistry was used to detect the expression levels of MAPK family kinases (p-P38, p-ERK, p-JNK) and Nrf2-Keap1 signaling pathway-related molecules in the placental tissues of each group. Data are presented as the mean ± SEM (A–F). *p < .05, **p < .01, ***p < .001, control group compared to the L-NAME group; ^###^*p* < .001, ^##^*p* < .01, ^#^*p* < .05. The comparison between the L-NAME+DMB group and the L-NAME group was performed by one-way ANOVA and Tukey’s multiple tests; *n* = 6.

Placental tissues of L-NAME-induced PE mice were collected to detect oxidative damage markers. The results revealed oxidative stress damage in the placenta of PE model mice, with increased MDA levels and decreased activity of antioxidant enzymes such as SOD and CAT, all of which were significantly ameliorated by targeted inhibition of TMAO with DMB (Figure 6a). We further examined the serum levels of inflammatory factors in the PE model mice and found that the serum levels of IL-6, TNF-α, and IL-1β had increased compared to those in the control group, and the levels of inflammatory factors were significantly enhanced after DMB-targeted inhibition of TMAO (Figure 6b). Furthermore, the expression levels of antioxidant proteins such as Nrf2, Keap1, and HO-1 were significantly reduced in the L-NAME model group, which was partially reversed by TMAO inhibition (Figure 6c–d). A significant increase in histone phosphorylation in the L-NAME model suggested further activation of the MAPK pathway in the PE model, whereas the activated signaling pathway was partially inhibited by DMB intervention (Figure 6e–f). Similar results were obtained by immunohistochemistry of key proteins (Figure 6g). In conclusion, targeted intervention with TMAO may alleviate symptoms in PE mice by reducing placental oxidative stress and inflammation.

### TMAO promotes oxidative stress and inflammatory injury and inhibits vascular endothelial cell migration and angiogenesis

**Figure 7.**
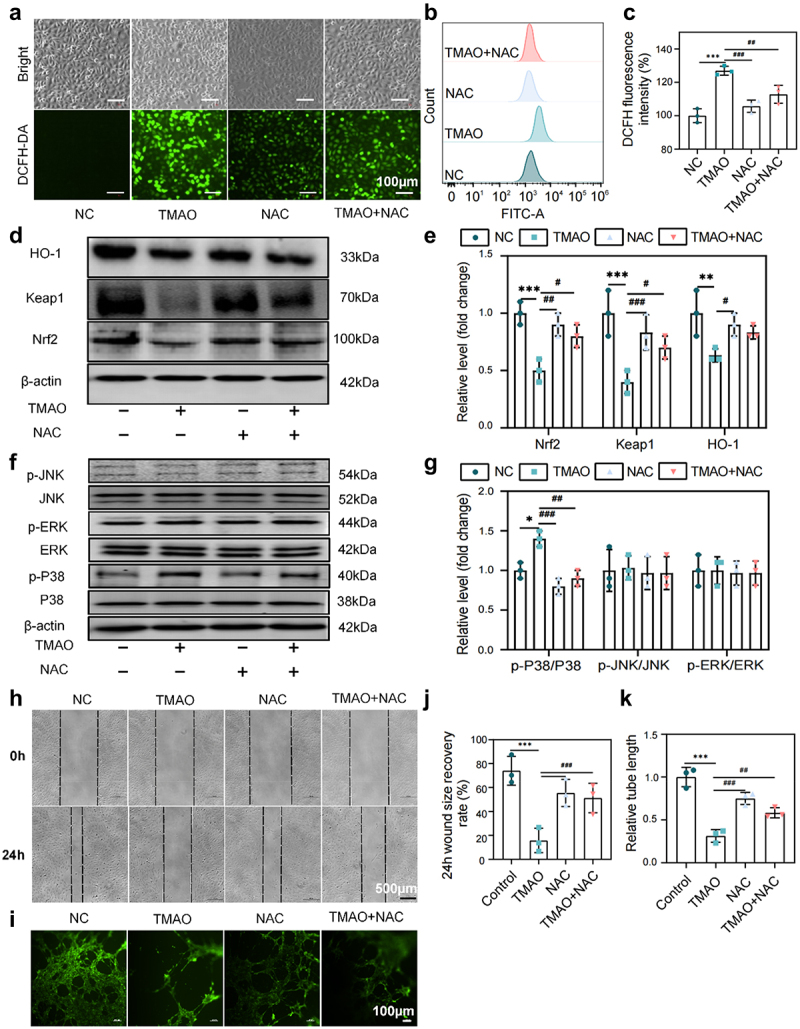
TMAO promotes oxidative stress and inflammatory injury and inhibits HUVEC migration and angiogenesis. (a) Fluorescence imaging was used to detect the intracellular reactive oxygen species (ROS) level in HUVECs after TMAO treatment. (b-c) flow cytometry was used to detect the intracellular ROS level of HUVECs after TMAO treatment. (d-e) expression levels of molecules related to the Nrf2-Keap1 signaling pathway. (f – g) expression levels of molecules associated with MAPK family kinase (P38, p-P38, ERK, p-ERK, JNK, p-JNK) signaling pathways. (h-j) TMAO affects the migration ability of HUVECs. (l-k) TMAO affects the angiogenic ability of HUVECs. Data are presented as the mean ± SEM. Differences between the mean values of the normally distributed data were analyzed using the wilcoxon rank sum test. *p < .05, **p < .01, ***p < .001, control group compared to the TMAO group; ###P < .001, ##P < .01, #P < .05, respectively, the comparison between the NAC, TMAO+NAC, and TMAO groups; n = 3.

To explore the cellular mechanism of TMAO in the progression of PE, we used TMAO-induced HUVECs to establish an oxidative stress injury model to mimic the oxidative stress of the PE placenta in vitro. Pre-experiments showed that treatment of HUVECs with 400 µM TMAO for 12 h had the most significant effect on inducing oxidative stress (Supplemental Fig. S7a-c). The ability of TMAO (400 µM, 12 h) to induce intracellular reactive oxygen species (ROS) production was measured by fluorescence microscopy and flow cytometry. The results revealed that the TMAO group had a significant increase in ROS levels compared to the control group, while the ROS scavenger N-acetyl-L-cysteine (NAC, a scavenger of ROS) pretreatment resulted in a significant reduction in intracellular ROS generation (Figure 7a–c). These results suggest that TMAO can induce oxidative stress damage by increasing intracellular ROS production. Additionally, TMAO activated the intracellular MAPK/Nrf2-Keap1 signaling pathway, with decreased levels of Nrf2, Keap1, and HO-1 protein and increased p38 phosphorylation in TMAO-treated cells. NAC treatment reversed the changes in the expression of proteins related to oxidative stress injury induced by TMAO (Figure 7d–g). Additionally, TMAO inhibited the migration and angiogenesis of HUVECs, and the inhibitory effects were reversed by NAC (Figure 7h–k). These results suggest that TMAO induces oxidative stress and inflammatory damage by activating the MAPKs/Nrf2-Keap1 pathway, which affects the migration and angiogenesis ability of HUVECs and promotes the progression of PE.

### TMAO promotes oxidative stress and inflammatory injury and inhibits the migration and invasion of placental trophoblast cells

**Figure 8.**
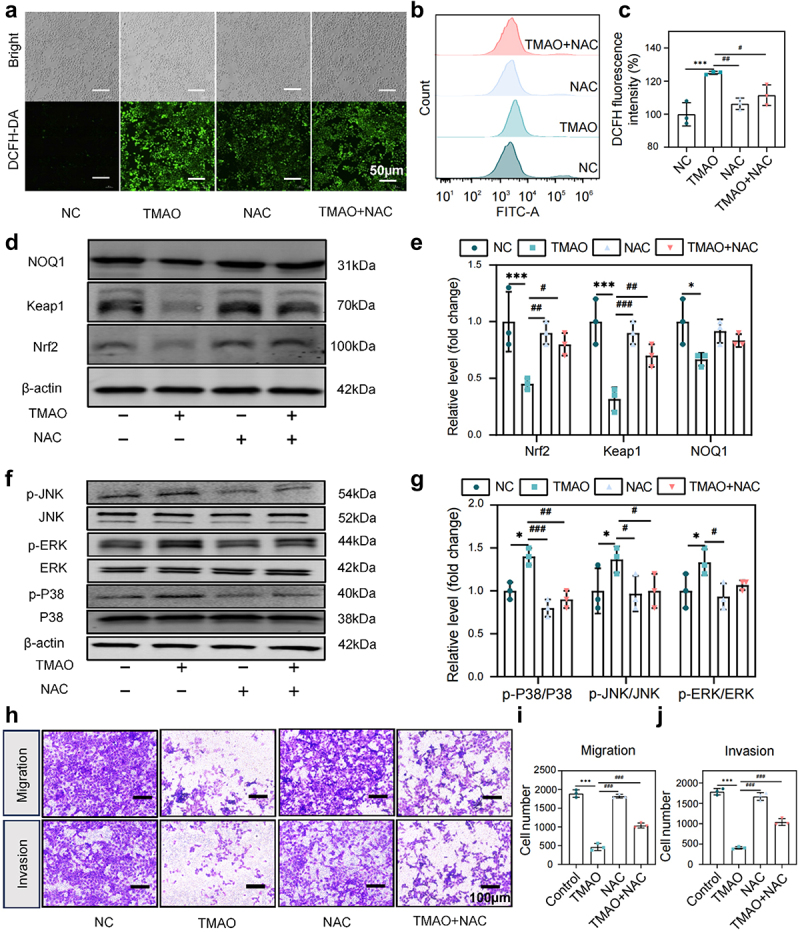
TMAO inhibits the migration and invasion of HTR-8/SVneo cells by activating the MAPK/Nrf2-Keap1 signaling pathway. (a) Fluorescence imaging was used to detect the intracellular ROS level in HUVECs after TMAO treatment. (b-c) flow cytometry was used to detect the intracellular ROS level of HUVEC after TMAO treatment. (d-e) expression levels of molecules related to the Nrf2-Keap1 signaling pathway. (f-g) expression levels of molecules associated with MAPK family kinase (P38, p-P38, ERK, p-ERK, JNK, p-JNK) signaling pathways. (h) TMAO affected the migration and invasion of HTR-8/SVneo cells. Data are presented as the mean ± SEM. Differences between the mean values of the normally distributed data were analyzed using the wilcoxon rank sum test. *p < .05, **p < .01, ***p < .001, control group compared to the TMAO group;^###^*p* < .001, ^##^*p* < .01, ^#^*p* < .05, respectively, comparison between the NAC, TMAO+NAC, and TMAO groups; *n* = 3.

Similarly, HTR-8/SVneo cells were used to mimic the placental oxidative stress induced by TMAO in PE. HTR-8/SVneo cells were treated with different concentrations of TMAO for different periods of time, with the results showing that TMAO at concentrations less than 2000 µM had no significant effect on the viability of HTR-8/SVneo cells within 48 h (Supplemental Fig. S7D-E). Optimal oxidative stress was induced in HTR-8/SVneo cells stimulated with 200 µm MTMAO for 48 h (Supplemental Fig. S7F). Flow cytometry and fluorescence microscopy showed that ROS levels in the TMAO group (200 µM, 48 h) were significantly increased compared to those in the control group and could be cleared by NAC (Figure 8a–c), indicating that TMAO promoted ROS production in HTR-8/SVneo cells. Additionally, TMAO activated the MAPKs/Nrf2-Keap1 signaling pathway, resulting in decreased protein expression levels of Nrf2, Keap1, and HO-1 and increased phosphorylation levels of p38, ERK, and JNK (Figure 8d–g), whereas the MAPKs/Nrf2-Keap1 signaling pathway was inhibited by NAC pretreatment. Additionally, TMAO inhibited the migration and invasion of HTR-8/SVneo cells (Figure 8h–j). These results suggest that TMAO induces oxidative stress by activating the MAPKs/Nrf2-Keap1 pathway, inhibits trophoblast cell migration and invasion, and affects the progression of PE.

## Discussion

In this study, the fecal intestinal flora of patients with PE was detected by 16S rRNA sequencing technology, which revealed dysbiosis and reduced diversity of flora, as well as significant increases in the abundance of *g_Klebsiella*, *g_Gemmobacter*, and *g_Ruegeria*, which produce TMAO, compared to healthy pregnant women. Gut microbiota imbalance represents a novel and recent discovery in the pathogenesis of PE.^18^ In 2020, Chen et al. reported that an imbalance of gut microbiota induces PE in pregnant women.^19^ Since then, Jin et al. examined the fecal gut microbiota of patients with PE and found a significant decrease in the abundance of the genus *Akkmermansia* compared to their healthy counterparts.^20^ This is consistent with the findings of Chen et al., who demonstrated increased abundances of *Fusobacterium*, *Veillonella*, *Clostridium perfringens*, and *Bulleidiamoore*i in fecal samples from pregnant women with PE, as well as decreased abundances of *Akkermansia*, *Coprococcucatus*, and *Faecalibacterium*.^19,21^ Moreover, in a mouse model of PE, extracellular vesicles derived from *Akkermansia muciniphila* promoted placental formation and alleviated the symptoms of PE.^22^ We also detected the placental microbiota of patients with PE and healthy pregnant women and found significant differences at the phylum level, but not at the genus level, which may be related to the barrier effect of the placenta. These results suggest that TMAO-producing bacteria may be the key flora responsible for the occurrence and development of PE, and reveal that gut microbiota and its metabolites have important diagnostic and therapeutic potential for PE. Correlation analysis showed that the abundance of *Gemmobacter* and *Ruegeria* was significantly and positively correlated with systolic and diastolic blood pressure levels, respectively, indicating that the abundance of TMAO-producing bacteria was closely related to the clinical symptoms of PE. However, the causal relationship between gut microbiota and PE and the related mechanisms need to be further verified and explored.

TMAO is an important metabolite of gut microbiota, and its precursors, including choline, betaine, creatinine, and L-carnitine, are converted to TMA by gut microbiota. TMA can then be oxidized to TMAO by FMOs. TMAO has been recognized as a potential risk factor for cardiovascular disease and other chronic diseases.^23–25^ PE is a pregnancy-specific hypertensive disease. Many studies have found that the serum TMAO level of pregnant women with PE is higher than that of healthy pregnant women.^10,13,26^ Furthermore, a clinical study by Wen et al. found that elevated TMAO levels are a risk factor for PE and are associated with systemic inflammation and endothelial dysfunction.^10^ Additionally, animal studies have further confirmed that elevated circulating TMAO levels promote vascular inflammation and oxidative stress, leading to endothelial dysfunction and hypertension.^27^ However, the role of TMAO in PE remains unclear. In this study, serum TMAO and its precursors were measured in healthy pregnant women and patients with PE using targeted metabolomics technology. The results showed that the serum TMAO levels were significantly higher in the PE group than in the healthy pregnant control group, as were the levels of its precursors, betaine, creatinine, and L-carnitine. Additionally, the levels of TMAO and its precursors were correlated with key clinical indicators of PE, including blood pressure, urea, and fetal birth weight, suggesting that TMAO and its precursors may be involved in the progression of PE. The ROC curve analysis showed that TMAO and its precursors could be used to diagnose PE, among which, creatinine had the highest sensitivity and specificity (AUC = 1, sensitivity = 100%, and specificity = 100%). Therefore, TMAO and its precursors represent novel biomarkers for the diagnosis of PE.

To explore the relationship between gut microbiota imbalance and PE, fecal microbiota from patients with PE were transplanted into antibiotic-depleted mice. Mice that had received fecal microbiota from patients with PE presented typical clinical phenotypes of PE, including elevated blood pressure and proteinuria during pregnancy, whereas the addition of a TMAO-specific inhibitor to FMT reversed the progression of PE and improved pregnancy outcomes. These results suggest that FMT from patients with PE can cause typical PE symptoms in mice, as well as adverse pregnancy outcomes and placental structural changes. By analyzing the cecal contents of mice, we observed gut microbiota dysbiosis in the PE-FMT-induced PE model. We also found that targeted intervention with TMAO could significantly improve the intestinal dysbiosis in the FMT group; the level of TMAO in the FMT group was significantly higher than that in the control group; and the level of TMAO decreased significantly after targeted inhibition of TMAO. Intestinal flora imbalance is an important cause of PE, in which TMAO-producing bacteria and elevated serum TMAO levels may be the key reasons for the occurrence and progression of PE.

To further clarify whether intervention with TMAO could prevent the progression of PE, we established an L-NAME-induced PE mouse model and tested the reversal effect of the TMAO inhibitor DMB on the symptoms of PE. We found that reducing TMAO levels significantly alleviated clinical symptoms and improved pregnancy outcomes and placental pathology in PE mice. These results suggest that TMAO has potential value as a new therapeutic target for PE. However, to date, the mechanism of action of TMAO in PE remains unclear. Studies have found that abnormal oxidative stress in vivo can cause vascular endothelial cell damage^28^ and placental trophoblast dysfunction,^3^ representing one of the key factors leading to PE. In the present study, we observed that TMAO inhibited the migration and angiogenesis of HUVECs and the migration and invasion of HTR-8/SVneo cells in vitro, and the inhibitory effects of TMAO were reversed by NAC. TMAO also promoted excessive oxidative stress in HUVECs and HTR-8/SVneo cells, with significantly increased production of intracellular ROS, consistent with the results of other similar studies.^29–31^ ROS is associated with the activation of redox-sensitive signal transduction, such as the mitogen-activated protein kinase (MAPK) pathway.^32^ We found that TMAO significantly upregulated the phosphorylation of p38, ERK, and JNK, and activated MAPK inflammation-related signaling pathways. The results showed that a TMAO-specific inhibitor reduced the progression of PE in the mouse placenta by activating the Nrf2/Keap1 signaling pathway. TMAO can inhibit the migration and angiogenesis of HUVECs and the migration and invasion of HTR-8/SVneo cells by inhibiting the Nrf2/Keap1 signaling pathway, and the inhibitory effect of TMAO can be reversed by NAC.

Taken together, the results of the current study suggest that TMAO and its producing bacteria are key to the progression of PE, and its mechanism of action is related to the activation of the MAPK/Nrf2-Keap1 signaling pathway, induction of oxidative stress injury, further inhibition of HUVEC migration and angiogenesis, and inhibition of HTR-8/SVneo cell migration and invasion. Targeted intervention with TMAO is an effective means of treating PE.

However, there are several limitations to our study that warrant discussion. First, the antibiotic depletion experiment used in this study has a certain limitation pertaining to the elimination rate of bacterial flora in mice. An FMT experiment using germ-free mice would further prove the pathogenic role of bacterial flora. Second, several strains of TMAO were identified in this study, and future studies on the intervention and mechanism of individual strains are needed to provide a basis for precise diagnosis and treatment. We explored the mechanism of TMAO by detecting the oxidative stress level and expression of related proteins in the MAPKs/Nrf2-Keap1 signaling pathway; however, future studies are required to establish the interaction of signaling proteins in the pathway to further clarify the molecular mechanism underlying the effects of TMAO on the development of PE. Such studies will be helpful to further explain the pathogenesis of PE and provide a more robust experimental basis for the discovery of new targets for the diagnosis and treatment of PE.

In this study, we identified the role and mechanism of gut microbiota and TMAO in the occurrence and development of PE by inducing oxidative stress and inflammation to promote vascular endothelial injury and trophoblast dysfunction. Our findings serve to provide novel ideas and a theoretical basis for the pathogenesis of PE, as well as an experimental basis for gut microbiota and TMAO as a new target for the clinical intervention of PE.

## Methods

### Clinical cohorts

Thirty-eight pregnant women with PE and 29 normal pregnant women who delivered in the Department of Obstetrics of the investigator’s hospital from October 2020 to June 2021 were selected as the research subjects. PE was diagnosed according to the American College of Obstetricians and Gynecologists (ACOG) criteria.^33^ Patients with new-onset hypertension (systolic and/or diastolic blood pressure ≥ 140/90 mmHg) combined with proteinuria or end-organ damage after 20 weeks of gestation were included in the PE group. Pregnant women with normal blood pressure levels during the entire pregnancy and no metabolic diseases or other pregnancy complications were included in the NP group. Patients were excluded if they had taken antibiotics or probiotics or had diarrhea or other gastrointestinal symptoms within 1 month before sampling. The demographic characteristics and clinical information of the subjects are presented in Supplementary Tables S2–4.

This study was approved by the ethics committee of our hospital (No. KY202011224). All of the subjects were informed of the nature of this study, and informed consent was obtained in accordance with the Declaration of Helsinki. All of the procedures were performed in accordance with relevant laws and institutional guidelines.

### Collection of clinical data and biological specimens

The clinical data of the two groups were collected, including age, height, pre-pregnancy body mass index (BMI), weight gain during pregnancy, blood pressure, marriage, and childbearing history. In parallel, the blood indices of pregnant women before delivery (e.g., white blood cell [WBC] count, hemoglobin [Hb], platelet, WBC ratio, total bile acid, urea/creatinine, activated partial thromboplastin time [APTT], D-dimer) and fetal ultrasound indicators (e.g., biparietal diameter, abdominal circumference, and S/D value) were uniformly derived in the hospital medical record system. Pregnancy outcomes, including gestational age at delivery and fetal birth weight, were recorded after delivery. Blood samples were centrifuged (3000 g, 4°C, 15 min) within 2 h at room temperature or within 4 h at 4°C. Serum samples were collected and stored at − 80°C. A fresh fecal sample (30 mg) was collected before delivery, and the placental tissue was collected after delivery and stored at − 80°C.

### 16S rRNA gene sequencing of the fecal and placental microbiota

Total genomic DNA was extracted from stool and placental tissue samples using a modified cetyltrimethylammonium bromide (CTAB) method. After DNA extraction, the V4 region of the 16S rDNA was amplified by PCR using universal primers under the following thermal cycling conditions. The Illumina Hiseq 2500 platform was used for amplicon sequencing. After the original data had been subjected to quality control steps, such as removing connectors, primers, and low-quality sequences using QIIME2 software, a Features table was generated to obtain ASV representative sequences, which were then annotated and compared in the database. Statistical tables of taxonomic annotation information of kingdom, phylum, class, order, family, genus, and species were obtained, and community composition, α diversity, β diversity, and functional prediction were analyzed.

### Detection of serum TMAO and its precursors by liquid chromatography-mass spectrometry (LC-MS)

A 20 μL volume was aspirated from each of the serum samples collected, before adding 10 μL TMAO internal standard solution and 750 μL 1% formic acid-acetonitrile solution. After vortexing for 30 s and centrifuging at 12,000 rpm for 5 min at 4°C, 500 μL of supernatant was taken and filtered through a 0.22-μm membrane. Subsequently, the filtrate was added to the detection flask and detected by LC-MS/MS. Based on the results, targeted quantification of TMAO and its precursors in the serum samples of PE and NP pregnant women was performed, and the relevant data were analyzed according to the quantitative results. OPLS-DA was used to avoid reducing the predictive ability of the model. The complexity of the model was reduced and the interpretation ability of the model was enhanced to maximize the differences between groups. Subsequently, hierarchical cluster analysis was performed using the relative values of TMAO and its precursors under different experimental conditions as the results are presented as heatmaps. The bar chart shows the dispersion of a set of data. Spearman correlation analysis was used to calculate the correlation coefficient (r) of non-normally distributed data; *r* values between 0.36 and 0.67 were considered moderate correlations, whereas values between 0.68 and 1.0 were considered strong correlations. A ROC curve was used to evaluate and screen potential biomarkers.

### RT-qPCR analysis

Total feces mRNA was extracted by using a Stool RNA Extraction Mini Kit (RNS 482, ONREW), followed by reverse transcription and real-time fluorescence quantification by using US Everbright® Inc. RT mix (R2020L) and SYBR Green qPCR Supermix (S2024L), respectively. Real-time fluorescence quantification and the amplification were performed on a Bioer Technology Fluorescence Quantitative Polymerase Chain Reaction Detection System (FQD-96A). The primer sequences for the target bacteria were as follows: universal bacteria forward: 5′- ACTCCTACGGGAGGCAGCAGT −3′, reverse: 5′- ATTACCGCGGCTGCTGGC −3′; *g_Klebsiella* forward: 5′-AGGCGGGTTTGTAAGTAGGG-3′, reverse: 5′-AGTATCAGTCCAGCGAGTCG-3′; *g_Gemmobacter* forward: 5′-GCATATCAATAAGCGGAGGAAAAG-3′, reverse: 5′-GGTCCGTGTTTCAAGACGG −3′; *g_Ruegeria* forward: 5′-ATATGAATATCCTCCTTAG-3′, reverse: 5′-TGTAGGCTGGAGCTGCTTCG- 3′.

### Construction of a PE mouse model using humanized fecal microbiota transplantation

This study was approved by the ethics committee of our hospital (No. NBU20220129). Female C57BL/6N mice (6–8 weeks old) were housed in specific pathogen-free facilities under a 12-h/12-h light-dark cycle. The sample size of pregnant mice used in this study was further validated using the “resource equation” method based on the law of diminishing returns. The grouping, measurements, and statistical phases were blinded, and the experimental mice were randomly assigned to three groups: the Control, FMT, and FMT+DMB groups with six mice each. Antibiotics (vancomycin 100 mg/kg, neomycin sulfate, metronidazole, and ampicillin 200 mg/kg) were administered by gavage once daily for 5 days to deplete the intestinal flora. Feces from 3 patients with PE were selected as donors in the FMT group to prepare the fecal microbiota solution. To prepare the fecal microbiota solution, fresh feces were filtered through a 200-µm screen to remove large particulate matter. After three filtrations, the samples were centrifuged at 6000 g for 20 min at 4°C. The sediment was collected, 10–15 ml of sediment was dispensed in sterile tubes, and 20–25 mL of saline was added, suspended in 10% sterile glycerol solution, and stored at − 80°C.

Fecal microbiota fluid from three PE donors was pooled and used as a single source of FMT in mice. After antibiotic depletion, the mice in the FMT group were administered fecal microbiota solution from PE donors by gavage for 3 consecutive days, and then three times a week for 8 consecutive weeks (59 days). Six weeks after the first FMT, all of the female mice were housed overnight with male mice at a 3:1 ratio, and pregnancy was confirmed by checking the vaginal plug. After pregnancy, the mice were continuously gavaged with fecal microbiota solution three times a week until the 17th day of pregnancy. Urine protein was measured once before FMT, blood pressure was measured weekly after FMT, urine protein was measured once before mating, and blood pressure was measured every 3 days after mating. Mouse urine was collected 1 day before dissection, and urine protein levels were measured. At the end of the experiment, serum was collected from each group, and the fetal mice and placenta were dissected and weighed.

### L-NAME induces a classic PE mouse model

Female C57BL/6N mice aged 6–8 weeks were randomly divided into three groups (*n* = 6 mice per group): control, L-NAME, and L-NAME+DMB groups. The mice were selected and housed overnight with male mice at a 3:1 ratio, and the day of vaginal plug shedding was defined as gestational day 0.5 (G0.5). From G10 onward, mice in L-NAME group received daily subcutaneous injections of 125 mg/kg.d.bw L-NAME (N5751, Sigma-Aldrich) for 8 days,^34^ while mice in the L-NAME+DMB group were treated with 1%DMB in drinking water from G13 until G17. Treatments were randomly allocated to the experimental units. Urine protein levels were measured once before mating, and blood pressure was measured daily from G10. The urine of G17 mice was collected 1 day before mating, and 24 h urine protein was detected. G18 mice were anesthetized with 1.5% pentobarbital (60 mg/kg), serum was collected, and the placentas of fetal mice were dissected and weighed.

### Intervention methods for TMAO-specific inhibitors in PE mouse models

The effects of TMAO on PE progression were investigated in both the FMT and L-NAME models. The experimental groups were as follows: normal control, FMT model, FMT+DMB, L-NAME model, and L-NAME+DMB. The mice in the DMB intervention group were treated with 1% DMB (5258, Sigma-Aldrich) in drinking water, and the normal control mice were treated with an equal volume of sterile PBS by gavage. Blood pressure changes were measured after mating, and urine was collected from mice before mating and dissection to measure 24 h urinary protein. At the end of the experiment, serum was collected from the mice in each group, and the fetus and placenta were dissected and weighed.

### Measurement of blood pressure and proteinuria in mice

A ZS-Z mouse tail artery noninvasive blood pressure measuring instrument (No. MADLAB-4C/501 H, Beijing Zhongshi) was used to measure the blood pressure of mice. At room temperature, the mouse was placed on the fixed device in a quiet state, the tail airbag was placed in the proximal end of the mouse tail, and the pulse transducer was placed on the abdomen of the mouse and slowly heated to 39°C to fully expand the blood vessels of the tail. The other end was connected to the computer through a physiological 4-channel signal acquisition instrument. When the pulse signal appeared as a regular waveform, the systolic and diastolic blood pressure values were recorded. Each mouse was measured every 3 min, and the average value was repeated three times. The 24-h urine of mice in each group was collected in a standard metabolic cage, and the urinary protein concentration was detected using a urine protein quantification kit (C035–2, Nanjing Jijieng Bioengineering Institute). The optical density was measured at 595 nm using an automatic quantitative microplate reader (Thermo Fisher Scientific, USA). The 24 h urinary protein was calculated using the following formula: 24 h urine protein = urine protein concentration × 24 h urine volume.

### Measurement of placental tissue oxidative stress levels and serum inflammatory factors

The contents of MDA (A003-1-2, Nanjing Jiancheng Bioengineering Institute) and the activity of SOD (A001-3-2, Nanjing Jiancheng Bioengineering Institute) and CAT (A007-1-1, Nanjing Jiancheng BioEngineering Institute) in placental tissue were detected using kits, referring to the manufacturer’s instructions.

The levels of proinflammatory cytokines, including IL-6, TNF-α, and IL-1β, were quantified in mouse serum samples using a commercial ELISA kit (Elabscience®, Wuhan, China) according to the manufacturer’s instructions. The optical density was measured at 450 nm using a fully automated quantitative microplate reader (Thermo Fisher Scientific, USA). The serum cytokine concentrations were calculated according to the manufacturer’s instructions.

### Histopathological observation of the placenta

The placenta and kidney tissues of the mice in each group were washed with sterile PBS and fixed with 4% paraformaldehyde (v/v) for 48 h. After dehydration, embedding, and sectioning, H&E staining was used to observe the structure and inflammatory response of the placenta and kidney tissues. The structure of the placenta implantation site was observed, the ratio between the two functional areas of the placenta (labyrinth and junctional zone) was calculated, and the infarct size of the placenta and the deposition of collagen in the placental labyrinth layer were observed.

### Cell lines and culture

HUVECs and HTR-8/SVneo cells were purchased from Shanghai Zhongqiao Xinzhou Technology Co., Ltd. HUVECs were cultured in Endothelial Cell Medium (ECM) (Scien Cell) medium supplemented with 10% fetal bovine serum (FBS) (Scien Cell) and 1% penicillin-streptomycin (Scien Cell). HTR-8/SVneo cells were cultured in RPMI 1640 (Gibco) medium supplemented with 10% fetal bovine serum (BI) and 1% penicillin-streptomycin (Gibco). The cells were cultured at 37°C in an incubator containing 5% CO2. After reaching a cell density of approximately 70%, the cells were passaged or used for plating experiments.

### TMAO-induced cell oxidative stress injury model

HUVECs and HTR-8/SVneo cells were treated with 400 µM and 200 µM TMAO for 12 h and 48 h, respectively, to replicate the oxidative stress injury model. A DCFH-DA fluorescent probe and flow cytometry were used to detect the levels of ROS in the cells to determine whether the oxidative stress injury model was successfully induced.

### Measurement of intracellular ROS levels

HUVECs and HTR-8/SVneo cells were seeded in 6-well plates (3 × 10^6^/well) and treated with different concentrations of TMAO (0, 200, 400, 800, 1600 µM) for 12 h and 48 h, respectively, followed by the addition of 10 µM DCFH-DA probe and incubation at 37°C for 20 min. Intracellular ROS levels were qualitatively and quantitatively analyzed using fluorescence microscopy and flow cytometry (BECKMAN CytoFlex S).

### Cell viability assay

The effect of TMAO on cell viability was detected using the CCK-8 assay (K1018, APExBIO). HUVECs and HTR-8/SVneo cells were seeded in 96-well plates (1 × 10^3^ cells/well) and cultured at 37°C for 24 h. The cells were then treated with different concentrations of TMAO (0, 100, 200, 300, 400, 600, 800, 1000, and 2000 µM) for 24 h. Subsequently, 10 μL CCK-8 solution was added to each well, and the incubation was continued for 2 h at 37°C. Absorbance at 450 nm was measured using a microplate reader (Bio-Rad Laboratories, Inc.).

### HUVEC migration assay

HUVECs were seeded in 6-well plate cell climbs at a density of 1 × 10^6^/well and cultured until 95% confluence. Adherent monolayers were scratched with a 200-µL pipette tip. The scratch was marked with a marker pen, and culture was continued by adding medium, TMAO, NAC, and TMAO+NAC. Debris and isolated cells were washed off with phosphate buffered saline (PBS), and scratch images at 0, 12, and 24 h were photographed and recorded using a light microscope (Olympus Corporation, Japan). The scratch area was measured using ImageJ software.

### HUVEC angiogenesis assay

For the angiogenesis assay, 50 µL of matrix glue was added to each well of the 96-well plate, before placing on ice and then incubating at 37°C for 0.5 h to allow the matrix gel to solidify. HUVECs were suspended in the desired medium at a concentration of 3 × 10^5^ cells/ml. Subsequently, 50 µL of the cell suspension was seeded on Matrigel and incubated for 4–8 h, before observing tube formation under an inverted microscope. The number of tubes was measured using ImageJ software.

### HTR-8/SVneo cell migration assay

HTR-8/SVneo cells were cultured in 24-well plates with medium, TMAO, NAC, and TMAO+NAC. Cells were digested 24 h later and suspended in serum-free medium. Next, 200 μL of cell suspension was added to the upper chamber of a Transwell chamber, and 800 μL of RPMI 1640 medium containing 20% FBS was added to the lower chamber, before placing in a cell incubator. The chambers were removed after 24 and 48 h of culture, the medium in the upper chamber was blotted dry, and the Matrigel and cells in the upper chamber were gently swabbed with cotton swabs and fixed with 4% paraformaldehyde for 30 min. This was followed by staining with 0.1% crystal violet solution for 15 min. After washing three times with PBS, the number of transmembrane cells was observed under a light microscope.

### HTR-8/SVneo cell invasion assay

At 4°C, Matrigel gel was diluted with serum-free cell medium (1:8), before evenly adding 60 μL to the upper chamber surface of the bottom membrane of the Transwell chamber. The mixture was incubated at 37°C for 3 h to polymerize the matrix gel into a film. The subsequent steps are shown in the previous method.

### Immunohistochemical staining

Placental tissues from mice in each group were fixed, dehydrated, embedded, sectioned, dewaxed, hydrated, and heated in sodium citrate buffer (10 mM, pH = 6.0) to obtain antigens. Endogenous peroxidase activity was eliminated by 3% aqueous hydrogen peroxide for 15 min, and off-target antigens were blocked with 10% (v/v) goat serum. Tissue sections were incubated overnight at 4°C with primary rabbit monoclonal antibodies against Nrf2, Keap1, HO-1, p-P38, p-JNK, and p-ERK proteins (antibody information is provided in Supplementary Table S5). Subsequently, the sections were incubated with a secondary rabbit antibody conjugated to horseradish peroxidase for 45 min at 37°C. Horseradish peroxidase activity was observed with a diamine benzidine solution, followed by counterstaining with hematoxylin. Finally, the sections were dehydrated and sealed with neutral gum. Images were acquired using an EVOS FL automated imaging system (Thermo Fisher Scientific, USA).

### Western blot

Proteins were extracted from cells and placental tissues using RIPA lysis buffer (APExBIO, USA) containing a mixture of protease and phosphatase inhibitors. Subsequently, equal numbers of proteins were separated by SDS-polyacrylamide gel electrophoresis and transferred to PVDF membranes. After blocking with 5% skim milk for 2 h at room temperature, the cells were incubated with primary antibodies against Nrf2, Keap1, HO-1, P38, JNK, ERK, p-P38, p-JNK, and p-ERK (see Supplementary Table S5 for antibody information) overnight at 4°C, before washing with 3‰ TBST. Subsequently, the membranes were incubated with HRP-labeled secondary antibodies for 1 h at room temperature. Finally, the bands were immersed in the enhanced chemiluminescence (ECL) substrate (Forde Bio, China) working solution for 1 min and then placed in the Syngene fully automated gel imaging analysis system. Protein expression was quantified using Image J.

### Statistical analyses

GraphPad Prism software (v.8.0.2) was used for statistical analysis. Each assay and experiment was performed in at least three biological replicates, and data are presented as the mean ± SEM. Student’s *t*-test was used to compare the differences between groups, one-way ANOVA was used to analyze the statistical difference between multiple groups, and Spearman’s statistic was used for correlation analysis. In the Figure, *p* < .05 indicates statistical significance (*p < .05, **p < .01, ***p < .001; ^#^*p* < .05, ^##^*p* < .01, ^###^*p* < .001). Additional materials and methods are described in the Methods.

## List of abbreviations

PE: Pre-eclampsia
NP: Normal pregnant women
TMAO: Trimethylamine N-oxide
FMT: Fecal microbiota transplantation
DMB: 3,3-Dimethyl-1-butanol
L-NAME: NG-Nitroarginine methyl ester
TMA: Trimethylamine
FMOs: Flavin monooxygenases
HUVEC: Human umbilical vein endothelial cell
HTR-8/SVneo: Human placental trophoblast cell
NP: Normal pregnant women
OPLS-DA: Orthogonal projections to latent structures discriminant analysis
LEfSe: Linear discriminant analysis effect size
LDA: Linear discriminant analysis
ROC: Receiver operating characteristic curve
AUC: Area under the curve
PBS: Phosphate buffered saline
SBP: Systolic blood pressure
H&E: Hematoxylin-eosin
DBP: Diastolic blood pressure
MDA: Malondialdehyde
SOD: Superoxide dismutase
CAT: Catalase
IL-6: Interleukin
TNF-α: Tumor necrosis factor
IL-1β: Interleukin
ROS: Reactive oxygen species
ACOG: American College of Obstetricians and Gynecologists
BMI: Body mass index
WBC: White blood cell
Hb: Hemoglobin
APTT: Activated partial thromboplastin time
FBS: Fetal bovine serum
ECL: Enhanced chemiluminescence

## Supplementary Material

Supplemental Material

## Data Availability

The data that support these findings of the study are available upon request from the corresponding authors.
