# Supplementary material for: Gut microbiota-derived trimethylamine N-Oxide: a novel target for the treatment of preeclampsia
Source: Gut Microbes. 2024 Feb 13;16(1):2311888. doi: 10.1080/19490976.2024.2311888 (PMC10868535; doi:10.1080/19490976.2024.2311888)
Supplement: Supplemental Material [file KGMI_A_2311888_SM2103.zip › Table S5.docx]

**Table S4 Antibody information**

| **Target antigen** | **Vendor or Source** | **Catalog #** | **Western Blot Working**  **concentration** | **IHC Working**  **concentration** |
| --- | --- | --- | --- | --- |
| Nrf2 | Proteintech | 1631-1-AP | 1:1000 | 1:3000 |
| Keap1 | Proteintech | 10503-2-AP | 1:1000 | 1:3000 |
| HO-1 | Proteintech | 10701-1-AP | 1:1000 | 1:2000 |
| NOQ1 | Proteintech | 11451-1-AP | 1:1000 |  |
| P38 | Cell Signaling Technology | 9212S | 1:1000 |  |
| ERK | Cell Signaling Technology | 9102S | 1:1000 |  |
| JNK | Cell Signaling Technology | 9252S | 1:1000 |  |
| p-P38 | Cell Signaling Technology | 9211S | 1:1000 | 1:3000 |
| p-ERK | Cell Signaling Technology | 9101S | 1:1000 | 1:500 |
| p-JNK | Cell Signaling Technology | 9251S | 1:1000 | 1:200 |
| β-Actin | Affinity Biosciences | #AF7018 | 1:5000 |  |
| Anti-Mouse IgG | Proteintech | SA00001-1 | 1:1000 |  |
| Anti-Rabbit IgG | Proteintech | SA00001-2 | 1:1000 |  |
