# Supplementary material for: Gut microbiota-derived trimethylamine N-Oxide: a novel target for the treatment of preeclampsia
Source: Gut Microbes. 2024 Feb 13;16(1):2311888. doi: 10.1080/19490976.2024.2311888 (PMC10868535; doi:10.1080/19490976.2024.2311888)
Supplement: Supplemental Material [file KGMI_A_2311888_SM2103.zip › Table S2.docx]

**Table S2 Clinical Baseline Characteristics of the PE and NP Groups**

| **Characteristic** | **NP（n=29）** | **PE（n=38）** | ***P-*Value** |
| --- | --- | --- | --- |
| Age (years) | 28.19±2.39 | 29.82±4.78 | 0.089^b^ |
| **Education background, n (%)** |  |  | 0.179^c^ |
| College degree or above | 16（55.17） | 27（71.05） |  |
| High school degree or below | 13（44.83） | 16（28.95） |  |
| **working condition, n (%)** |  |  | 0.072^c^ |
| Housework and unemployment | 12（41.38） | 8（21.05） |  |
| Office clerk office clerk | 17（58.62） | 30（78.95） |  |
| Height（cm） | 163.14±5.11 | 161.16±5.04 | 0.118^a^ |
| Pre-pregnancy BMI (kg/m^2^) | 22.02±2.90 | 23.08±3.83 | 0.294^b^ |
| Weight gain during Pregnancy（kg） | 14.35±4.12 | 14.54±3.84 | 0.843^a^ |
| Fundal height（cm） | 34.59±2.47 | 33.71±3.14 | 0.180^b^ |
| Abdominal girth（cm） | 101.86±5.75 | 102.03±5.74 | 0.908^a^ |
| Gravidity | 1.97±1.18 | 2.03±1.37 | 0.854^b^ |
| **Parity, n (%)** |  |  | 0.407^b^ |
| Primiparous | 17 (0.59) | 26 (0.71) |  |
| Multiparous | 12 (0.41) | 12 (0.29) |  |

Note: ^a^ is from Student's t test;

^b^ is from the Wilcoxon rank sum test；

^c^ is from the chi-square test or Fisher's exact test;
