# Supplementary material for: Gut microbiota-derived trimethylamine N-Oxide: a novel target for the treatment of preeclampsia
Source: Gut Microbes. 2024 Feb 13;16(1):2311888. doi: 10.1080/19490976.2024.2311888 (PMC10868535; doi:10.1080/19490976.2024.2311888)
Supplement: Supplemental Material [file KGMI_A_2311888_SM2103.zip › Table S1.docx]

Table S1 Trimethylamine oxide precursor and intestinal flora traceability

| NAME |  | Betaine | Choline | Creatinine | Trimethylamine | Trimethylamine N-oxide |
| --- | --- | --- | --- | --- | --- | --- |
| KEGG_ID |  | C00719 | C00114 | C00791 | C00565 | C01104 |
| gut.y | Microbial metabolic production | Y | Y | Y | Y | Y |
| hsa.y | Host metabolic production | Y | Y | Y | Y | Y |
| eae. | k_Bacteria;p_Gammaproteobacteria - Enterobacteria;g_Klebsiella;s_Klebsiella aerogenes KCTC 2190 | Y | Y | Y | Y | Y |
| geh. | k_Bacteria;p_Alphaproteobacteria;g_Gemmobacter;s_Gemmobacter aquarius | Y | Y | Y | Y | Y |
| rua. | k_Bacteria;p_Alphaproteobacteria;g_Ruegeria;s_Ruegeria sp. AD91A | Y | Y | Y | Y |  |
