# Supplementary material for: Gut microbiota-derived trimethylamine N-Oxide: a novel target for the treatment of preeclampsia
Source: Gut Microbes. 2024 Feb 13;16(1):2311888. doi: 10.1080/19490976.2024.2311888 (PMC10868535; doi:10.1080/19490976.2024.2311888)
Supplement: Supplemental Material [file KGMI_A_2311888_SM2103.zip › Supplementary Figures.docx]

**
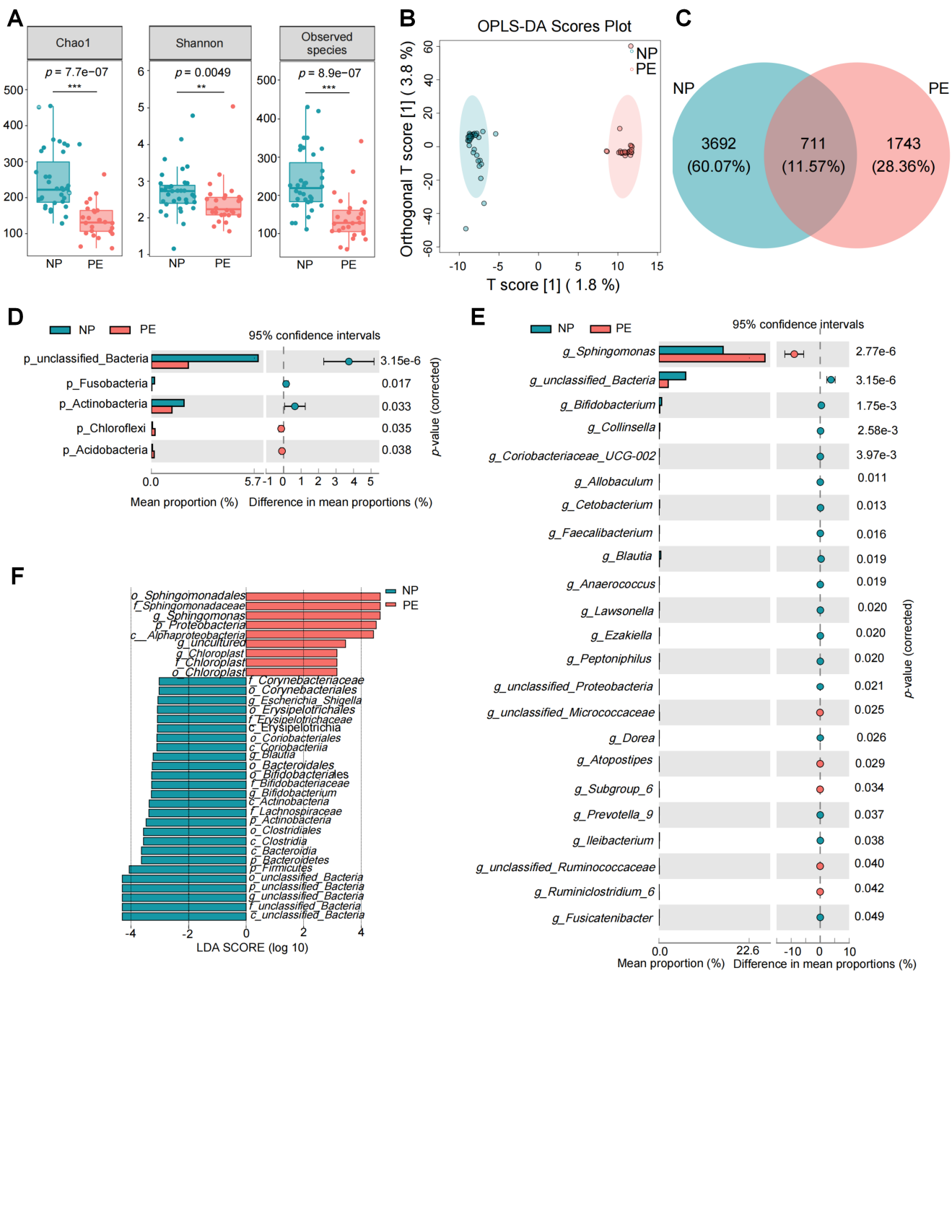
**

**Fig. S1 Dysbacteriosis is present in the placentas of patients with PE.** (A) Comparison of the α diversity of the placental microbiota between normal pregnant women (NP) and patients with PE. (B) Comparison of the β-diversity of the placental microbiota between NP group and PE group. (C) Community analysis was performed using Venn diagrams to compare the number of OperationalTaxonomic Units (OTUs) for gut microbes unique to each sample (group) and the number of common OTUs between the PE and NP group samples. (D) The relative abundance of the top 10 placental bacteria in the PE group was significantly different from that in the NP group at the phylum level. (E) There was no significant difference in the relative abundance of the top 10 placental bacteria between the PE and NP groups at the genus level. (F) Bar plot showing the linear discriminant analysis (LDA) value distribution of significantly different species, showing the significantly enriched species and their importance degree in the two groups. Statistical analysis was performed using the *t*-test. **P* < 0.05, ***P* < 0.01, ****P* < 0.001, NP group: n = 35; PE group: n = 26.


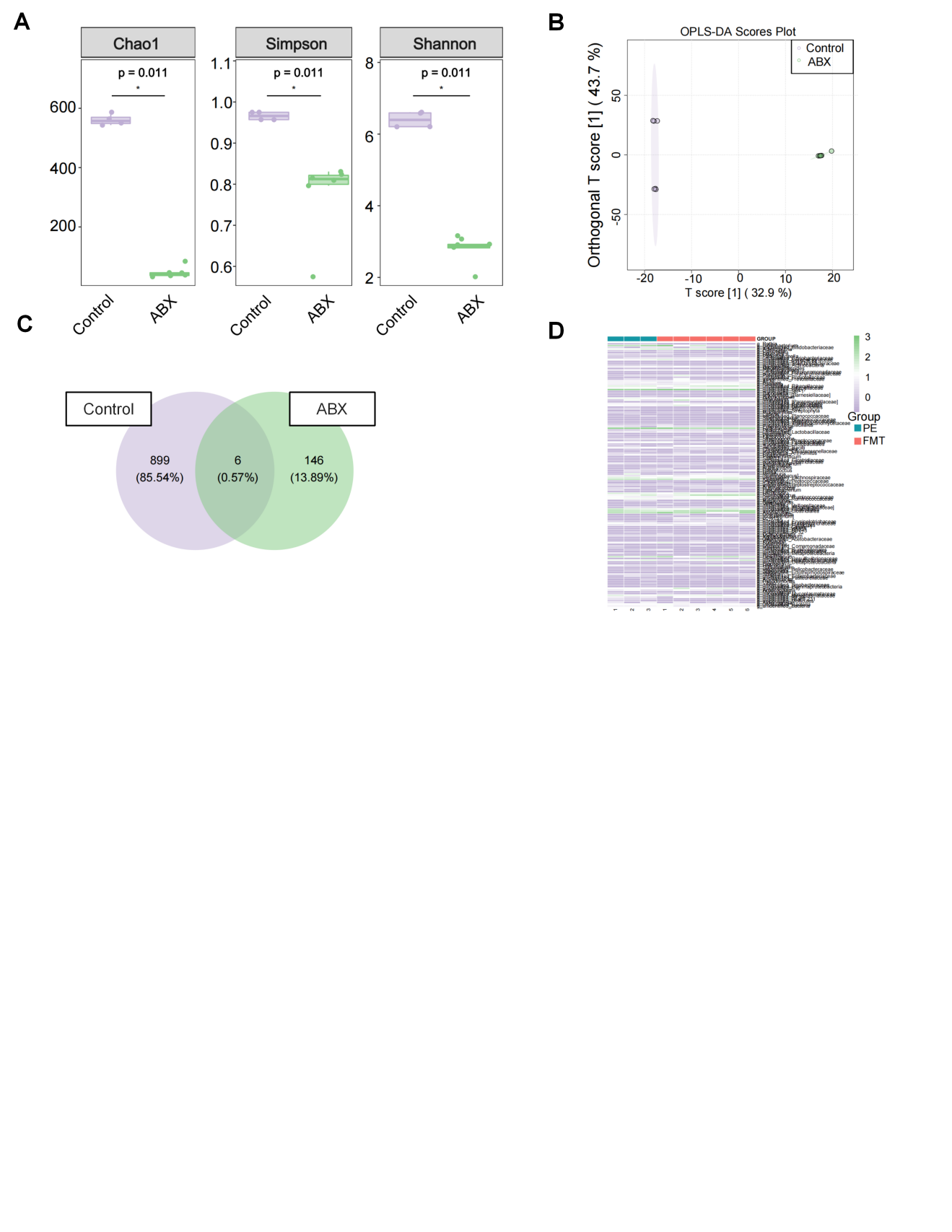


**Fig. S2 Antibiotic depletion and gut microbiota colonization in recipient mice.** (A) Comparison of the α diversity of the gut microbiota in mice before (Control) and after (ABX) antibiotic depletion (n = 6). (B) Comparison of the β diversity of the gut microbiota between Control and ABX groups. (C) Community analysis using Venn diagram to compare the number of specific flora or shared OTU between Control and ABX groups. (D) Heat map comparing the composition of gut microbiota between recipient mice and PE patient donors. Data are presented as mean ±SEM. Statistical analysis was performed using the *t*-test. **P* < 0.05, ***P* < 0.01, ****P* < 0.001.


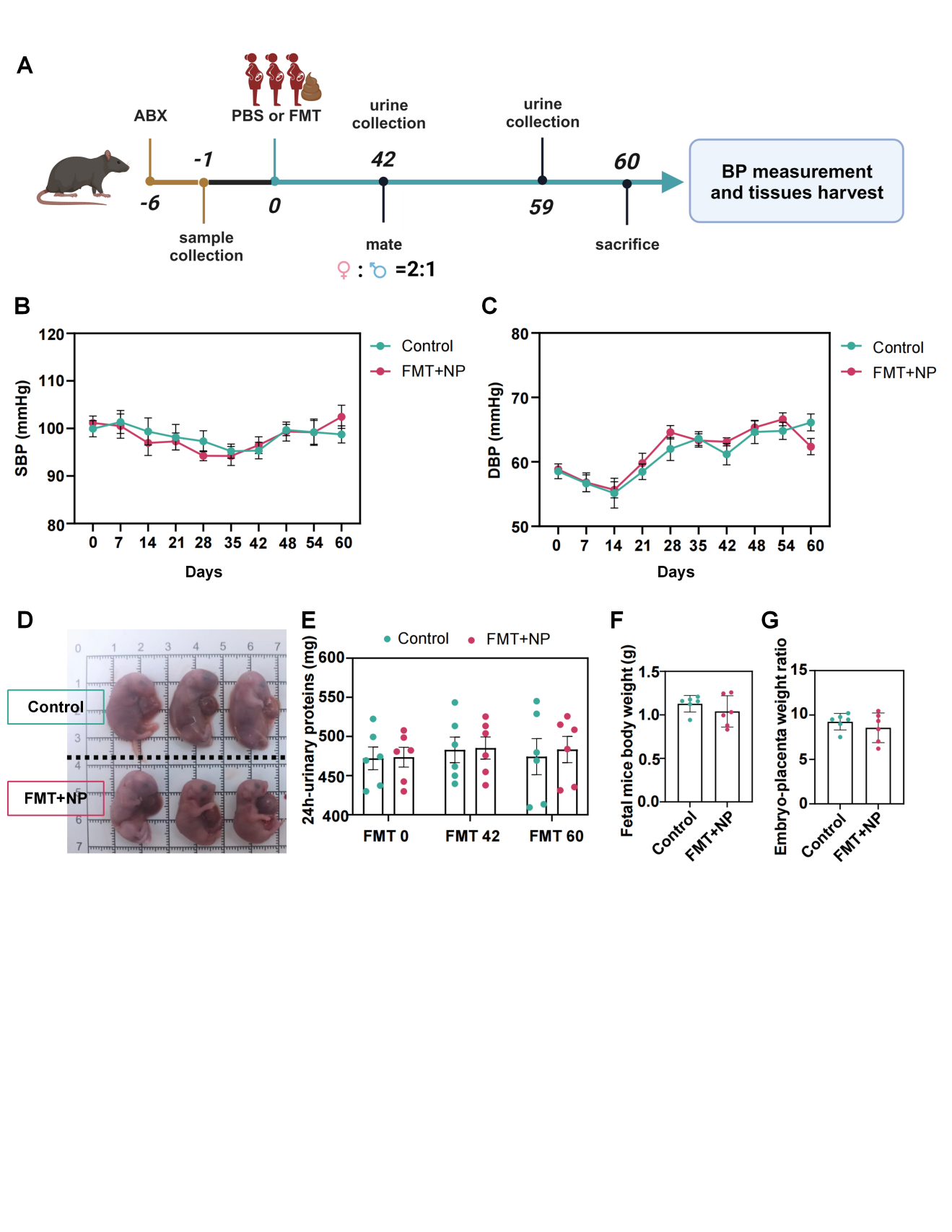


**Fig. S3 Clinical phenotypes and pregnancy outcomes did not differ significantly between the NP-FMT and control groups.** (A) NP-FMT-intervention protocol. (B) Overall photographs of the fetal mice and placentas in each group. (C) Dynamic changes and comparisons of the systolic blood pressure (SBP) of mice in each group. (D) Dynamic changes and comparison of the diastolic blood pressure (DBP) of mice in each group. (E) Twenty-four-hour urinary protein content before FMT, on day 42 of FMT (pre-pregnancy), and on day 59 of FMT (day 17 of pregnancy). (F) Mean weight of fetal mice. (G) Placental efficiency was assessed by the ratio of fetal mice weight to placental weight. (H) Data are expressed as the mean ± SEM, n = 6. **P* < 0.05, ***P* < 0.01, and ****P* < 0.001, NP-FMT group vs. control group.


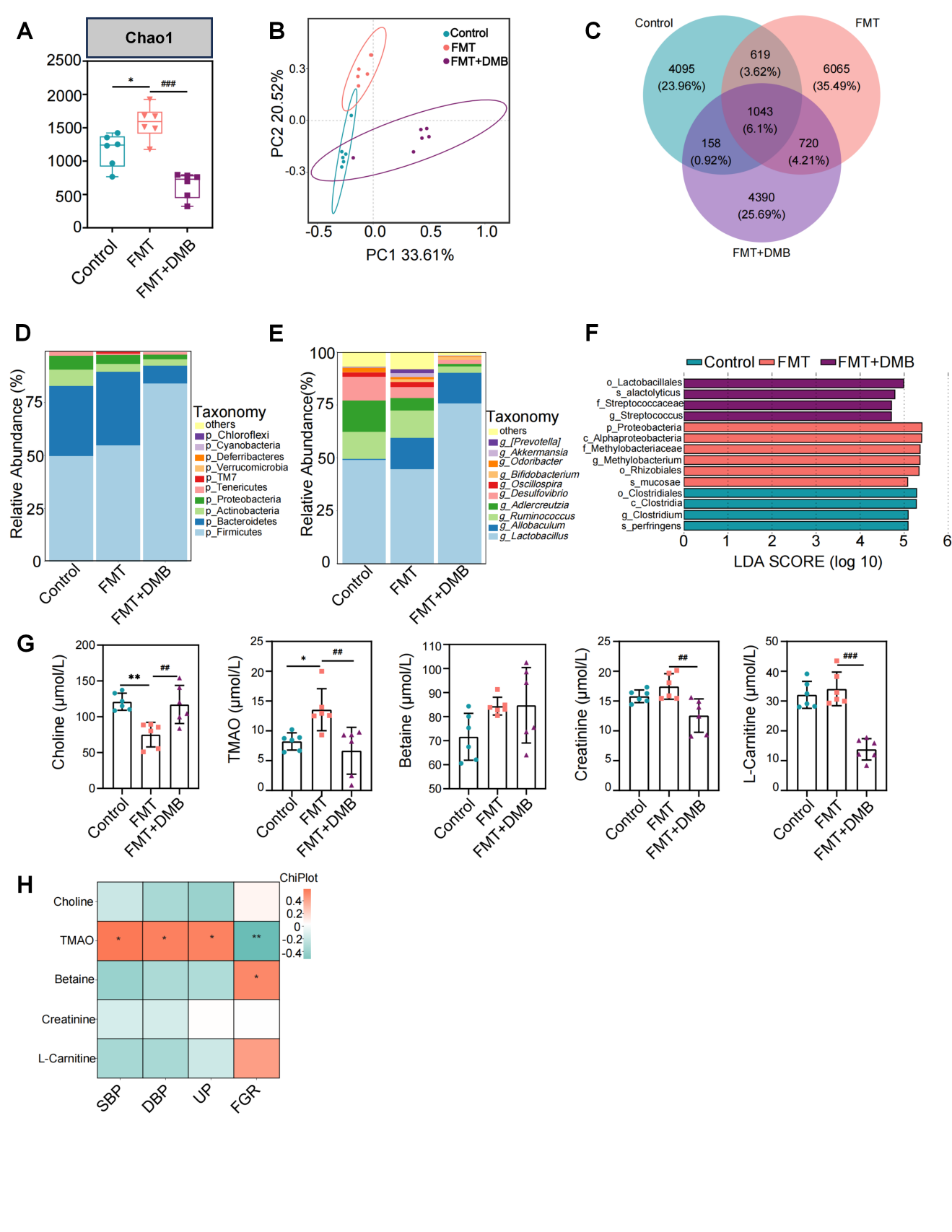


**Fig. S4 Targeted inhibition of TMAO corrects FMT-induced imbalance in the gut microbiota and the level of TMAO.** (A) Chao1 index in the fecal microbiota. (B) Principal coordinate analysis (PCoA) plot of the GM based on Bray-Curtis metrics. (C) Venn diagram showing the overlap of OTUs found in the three groups of gut microbiota. (D) Top 10 microbial taxa found at the phylum level. (E) Top 10 microbial taxa found at the genus level. (F) Histogram of distributions based on LDA (LDA > 4). (G) Serum levels of TMAO and its precursors in mice; (G) Correlation analysis between serum TMAO and its precursors and key clinical indicators of PE mice. n = 6. Data are presented as the mean ± SEM. Significant differences based on one-way ANOVA and Tukey’s multiple tests: **P* < 0.05, ***P* < 0.01, ****P* < 0.001, control group compared to the FMT group; ^###^*P* < 0.001, ^##^*P* < 0.01, ^#^*P* < 0.05, FMT+DMB group compared to the FMT group.SBP: Systolic blood pressure, DBP: Diastolic blood pressure,UP:urinary protein,FGR: fetal growth restriction.


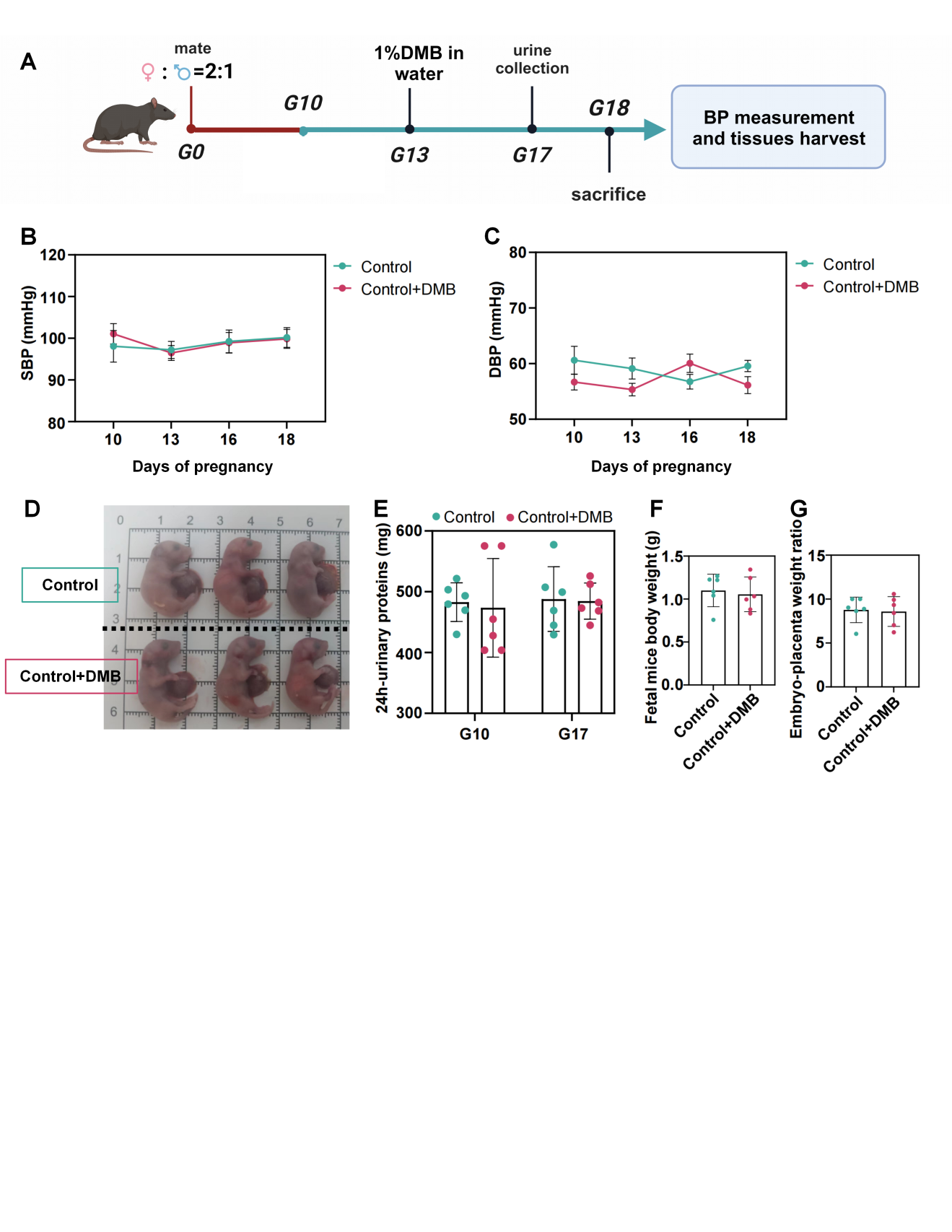


**Fig. S5 Clinical phenotypes and pregnancy outcomes did not differ significantly between the control+DMB and control groups.** (A) DMB-intervention protocol. (B) Overall photographs of fetal mice and placentas in each group. (C) Dynamic changes and comparisons of the systolic blood pressure (SBP) of mice in each group. (D) Dynamic changes and comparison of the diastolic blood pressure (DBP) of mice in each group. (E) Twenty-four-hour urinary protein content on day 17 of pregnancy. (F) Mean weight of fetal mice. (G) Placental efficiency was assessed by the ratio of fetal mice weight to placental weight. Data are expressed as the mean ± SEM, n = 6. **P* < 0.05, ***P* < 0.01, and ****P* < 0.001, control+DMB group vs. control group.

**
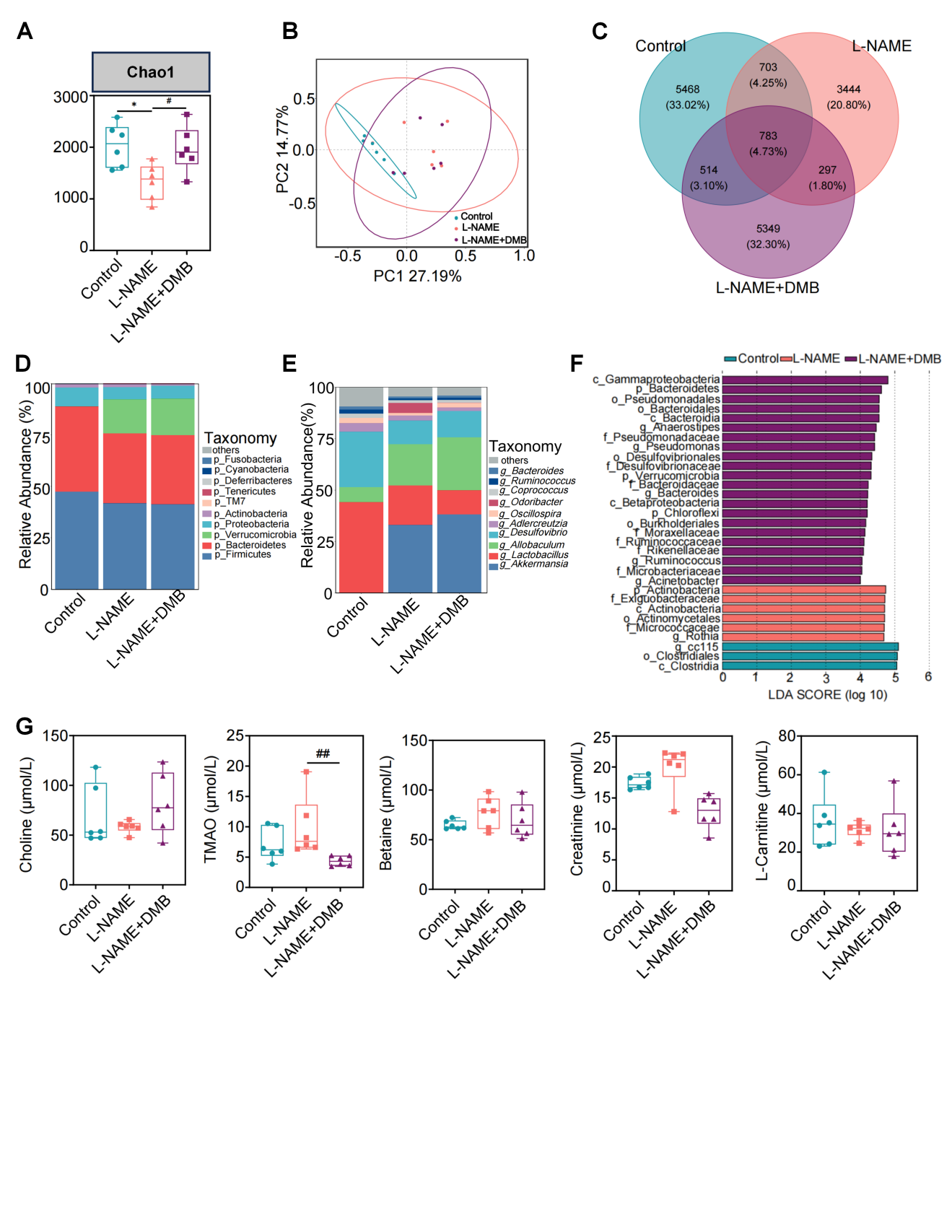
**

**Fig. S6 Targeted inhibition of TMAO corrects the L-NAME-induced imbalance in the gut microbiota and the level of TMAO.** (A) Chao1 index in the fecal microbiota. (B) Principal coordinate analysis (PCoA) plot of the gut microbiota based on Bray-Curtis metrics. (C) Venn diagram showing the overlap of OTUs found in the three groups of gut microbiota. (D) Top ten microbial taxa found at the phylum level. (E) Top ten microbial taxa found at the genus level. (F) Histogram of distributions based on LDA (LDA > 4). (G) Serum levels of TMAO and its precursors in mice; n = 6. Data are presented as the mean ± SEM. Significant differences based on one-way ANOVA and Tukey’s multiple tests: **P* < 0.05, ***P* < 0.01, ****P* < 0.001, control group compared to the L-NAME group; ^###^*P* < 0.001, ^##^*P* < 0.01, ^#^*P* < 0.05, L-NAME+DMB group compared to the L-NAME group.


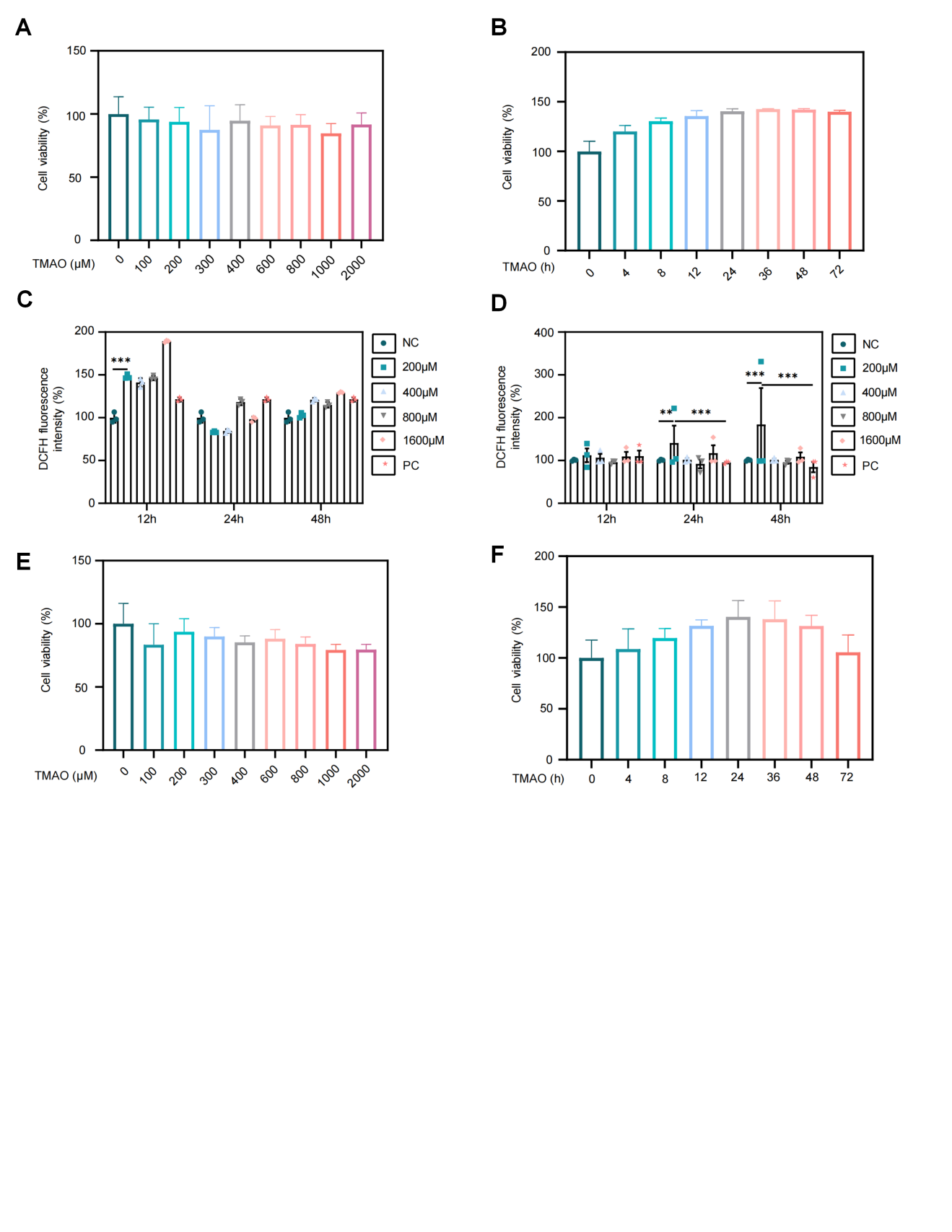


**Fig. S7 Exploration of HUVEC viability and oxidative stress model in HTR-8/SVneo cells.** (A) Effects of different concentrations of TMAO on HUVEC proliferation. (B) Effect of TMAO on HUVEC proliferation at different time points. (C) Relative levels of ROS produced by TMAO-induced oxidative stress at different time points (12 h, 24 h, 48 h) in HUVECs. (D) Relative levels of ROS produced by TMAO-induced oxidative stress in HTR-8/SVneo cells at different time points (12 h, 24 h, 48 h). (E) Effects of different concentrations of TMAO on the proliferation of HTR-8/SVneo cells. (F) Effects of TMAO on the proliferation of HTR8/SVneo cells at different time points. (F) Relative levels of ROS produced by TMAO-induced oxidative stress in HTR-8/SVneo cells at different times (12 h, 24 h, 48 h). Data are presented as the mean ± SEM. Differences between the mean values of the normally distributed data were analyzed using the Wilcoxon rank sum test. **P* < 0.05, ***P* < 0.01, ****P* < 0.001, control group compared to the TMAO group; ^###^*P* < 0.001, ^##^*P* < 0.01, ^#^*P* < 0.05, respectively, the comparison between the NAC, TMAO+NAC, and TMAO groups; n = 3.
