# Supplementary material for: Gut microbiota-derived trimethylamine N-Oxide: a novel target for the treatment of preeclampsia
Source: Gut Microbes. 2024 Feb 13;16(1):2311888. doi: 10.1080/19490976.2024.2311888 (PMC10868535; doi:10.1080/19490976.2024.2311888)
Supplement: Supplemental Material [file KGMI_A_2311888_SM2103.zip › Table S4.docx]

**Table S3 The prenatal blood and fetal ultrasound indexes of PE and NP groups were compared**

| **[clinical index](javascript:;)** | | **NP（n=29）** | **PE（n=38）** | ***P* Value** |
| --- | --- | --- | --- | --- |
| **[blood routine examination](javascript:;)** | |  |  |  |
|  | [white blood cell](javascript:;)(*10^9/L) | 10.83±3.21 | 8.81±2.28 | 0.002^b^** |
|  | [hemoglobin](javascript:;)(g/L) | 127.21±12.33 | 125.89±12.11 | 0.664^a^ |
|  | [neutrophilic granulocyte percentage](javascript:;)  (%) | 78.41±6.31 | 74.03±6.50 | 0.007^a^ ** |
|  | [blood platelet](javascript:;)(*10^9/L) | 191.07±46.64 | 191.95±52.87 | 0.894^b^ |
|  | CRP(mg/L) | 5.47±5.68 | 4.12±2.67 | 0.711^b^ |
| **[blood biochemistry](javascript:;)** | |  |  |  |
|  | [albumin](javascript:;)(g/L) | 37.85±6.40 | 35.24±3.63 | 0.071^b^ |
|  | [globulin](javascript:;)(g/L) | 26.87±3.33 | 26.93±3.62 | 0.940^a^ |
|  | [ratio of albumin to globulin](javascript:;) | 1.38±0.13 | 1.32±0.20 | 0.154^b^ |
|  | glutamic-pyruvic transaminase (U/L) | 9.48±3.21 | 12.00±8.28 | 0.218^b^ |
|  | [glutamic oxalacetic transaminase](javascript:;)(U/L) | 19.69±3.52 | 20.79±8.67 | 0.914^b^ |
|  | AST/ALT | 2.21±0.52 | 1.97±0.70 | 0.038^b^ * |
|  | TBA(μmol/L) | 2.69±1.35 | 4.46±2.59 | ＜0.001^b^ *** |
|  | Urea(mmol/L) | 3.26±0.75 | 4.02±1.02 | ＜0.001^a^ *** |
|  | creatinine (μmol/L) | 46.63±6.07 | 52.50±12.58 | 0.154^b^ |
|  | U/C | 0.07±0.02 | 0.08±0.02 | 0.045^b^ * |
|  | lithic acid(μmol/L) | 326.90±75.77 | 368.03±96.42 | 0.050^b^ |
|  | TB(μmol/L) | 8.78±2.91 | 7.96±3.32 | 0.126^b^ |
|  | DB(μmol/L) | 1.47±0.73 | 1.39±0.71 | 0.586^b^ |
|  | UB(μmol/L) | 7.30±2.31 | 6.57±2.97 | 0.096^b^ |
| **Blood clotting function** | |  |  |  |
|  | PT (s) | 11.06±0.59 | 11.03±0.42 | 0.827^a^ |
|  | APTT (s) | 26.90±1.57 | 26.56±1.81 | 0.425^a^ |
|  | TT (s) | 16.66±1.39 | 15.59±1.90 | 0.043^b^ * |
|  | Plasma fibrinogen assay(g/L) | 3.77±0.62 | 3.71±0.93 | 0.784^a^ |
|  | D-D | 603.83±339.51 | 534.22±364.06 | 0.526^b^ |
|  | Determination of fibrin (original) degradation products (μg/ml) | 7.50±3.67 | 6.63±3.64 | 0.355^b^ |
| **Fetal ultrasound** | |  |  |  |
|  | BPD(mm) | 93.45±3.61 | 89.90±4.73 | ＜0.001^b^ *** |
|  | head circumference (mm) | 334.31±11.22 | 325.05±15.49 | 0.006^a^ ** |
|  | abdominal perimeter (mm) | 336.31±19.07 | 319.56±22.21 | 0.002^a^ ** |
|  | femur length (mm) | 71.52±3.52 | 68.54±4.10 | 0.002^b^ ** |
|  | S/D | 2.22±0.33 | 2.34±0.34 | 0.091^b^ |
|  | RI | 0.54±0.06 | 0.56±0.06 | 0.086^a^ |

Note: ^a^ is from Student's t test;

^b^ is from the Wilcoxon rank sum test；

^c^ is from the chi-square test or Fisher's exact test;

**P*＜0.05，***P*＜0.01，****P*＜0.001
