# Supplementary material for: Gut microbiota-derived trimethylamine N-Oxide: a novel target for the treatment of preeclampsia
Source: Gut Microbes. 2024 Feb 13;16(1):2311888. doi: 10.1080/19490976.2024.2311888 (PMC10868535; doi:10.1080/19490976.2024.2311888)
Supplement: Supplemental Material [file KGMI_A_2311888_SM2103.zip › Table S3.docx]

**Table S3 Clinical presentation and pregnancy outcomes of the PE and NP Groups**

| **Presentation and outcomes** | **NP（n=29）** | **PE（n=38）** | ***P-*Value** |
| --- | --- | --- | --- |
| SBP on admission（mmHg） | 120.10±9.58 | 147.42±15.70 | ＜0.001^b^ *** |
| DBP on admission（mmHg） | 74.07±6.76 | 94.39±11.16 | ＜0.001^b^ *** |
| Gestational age at delivery (weeks) | 39.43±0.83 | 37.80±1.98 | ＜0.001^b^*** |
| **Delivery mode n（%）** |  |  | ＜0.001^c^ *** |
| Caesarean section | 6 (20.69) | 27 (71.05) |  |
| vaginal delivery | 23 (79.31) | 11 (28.95) |  |
| **Gender of the newborn n (%)** |  |  | 0.836^c^ |
| male | 13 (44.8) | 18 (47.4) |  |
| female | 16 (55.2) | 20 (52.6) |  |
| **Delivery outcome** |  |  |  |
| Neonate birth weight（kg） | 3.46±0.43 | 2.90±0.64 | ＜0.001^a^ *** |
| Blood loss at 2 hours postpartum（ml） | 256.90±49.02 | 338.03±183.51 | 0.174^b^ |

Note: ^a^ is from Student's t test;

^b^ is from the Wilcoxon rank sum test；

^c^ is from the chi-square test or Fisher's exact test;
